# Site-specific ERα phosphorylation determines sex-dependent metabolic, reproductive, and body-compositional phenotypes in mice

**DOI:** 10.1016/j.isci.2025.114112

**Published:** 2025-11-19

**Authors:** Binghao Zou, Jarvis Williams, Madeleine B. Landau, Weiqiang Lin, Sallie Fell, Ziqi Yang, MaryJane Jones, Robert Blair, Chad H. Steele, Pratik Khare, Cissy Zhang, Anne Le, Muralidharan Anbalagan, Brian G. Rowan

**Affiliations:** 1 Department of Structural and Cellular Biology, Tulane University School of Medicine, New Orleans, LA, USA; 2 Tulane Center for Biomedical Informatics and Genomics, Deming Department of Medicine, Tulane University School of Medicine, New Orleans, LA, USA; 3 Department of Microbiology and Immunology, Tulane University School of Medicine, New Orleans, LA, USA; 4 Department of Pathology and Laboratory Medicine, Tulane University School of Medicine, New Orleans, LA, USA; 5 Gigantest, Baltimore, MD, USA; 6 Department of Chemical and Biomolecular Engineering, Johns Hopkins University Whiting School of Engineering, Baltimore, MD, USA

**Keywords:** cell biology, metabolomics, transcriptomics

## Abstract

Estrogen receptor alpha (ERα) phosphorylation regulates receptor activity and tissue-specific gene expression. We generated serine (S) to alanine (A) phosphorylation-deficient knock-in mice targeting two conserved ERα sites, S171 and S216, to examine their physiological roles. ERα S216A females were subfertile, with ∼30% smaller litters and diminished uterine growth in response to estradiol (E2). Single-cell spatial transcriptomics revealed a disrupted E2-regulated transcriptome in the myometrium. Metabolic profiling revealed the suppression of glycolytic and redox pathways in ERα S216A mice, with males exhibiting reduced adiposity and increased lean mass. Skeletal analysis revealed opposing effects: ERα S216A females exhibited reduced femoral bone density, while ERα S171A females showed an increase. These data demonstrate critical roles for site-specific ERα phosphorylation in modulating receptor levels and activity, as well as gene expression, which have a profound impact on murine body composition, fertility, and metabolism.

## Highlights

• Site-specific ERα phosphorylation regulates sex-dependent physiology in mice
• S171A and S216A mutations differentially alter ERα protein across tissues
• S216A females show subfertility and blunted uterine response to estradiol
• Spatial transcriptomics reveal ECM and smooth muscle pathway disruption

## Graphical abstract

**Figure:**
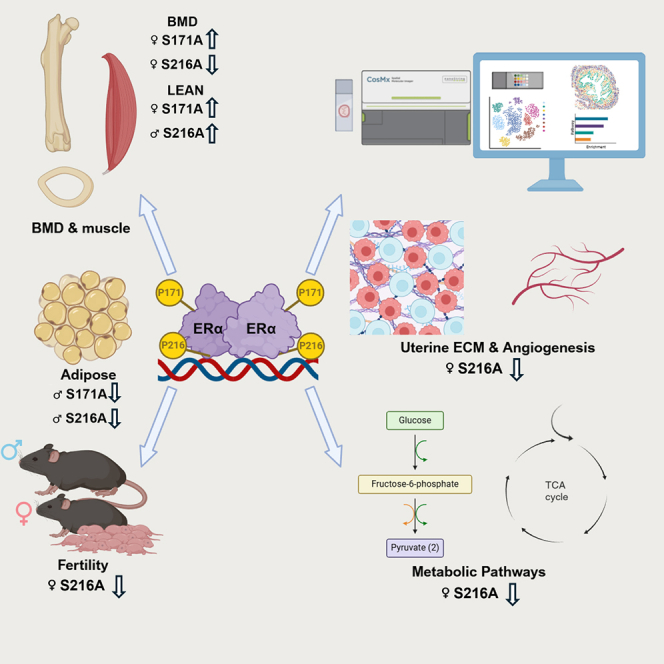

## Introduction

**Figure 1:**
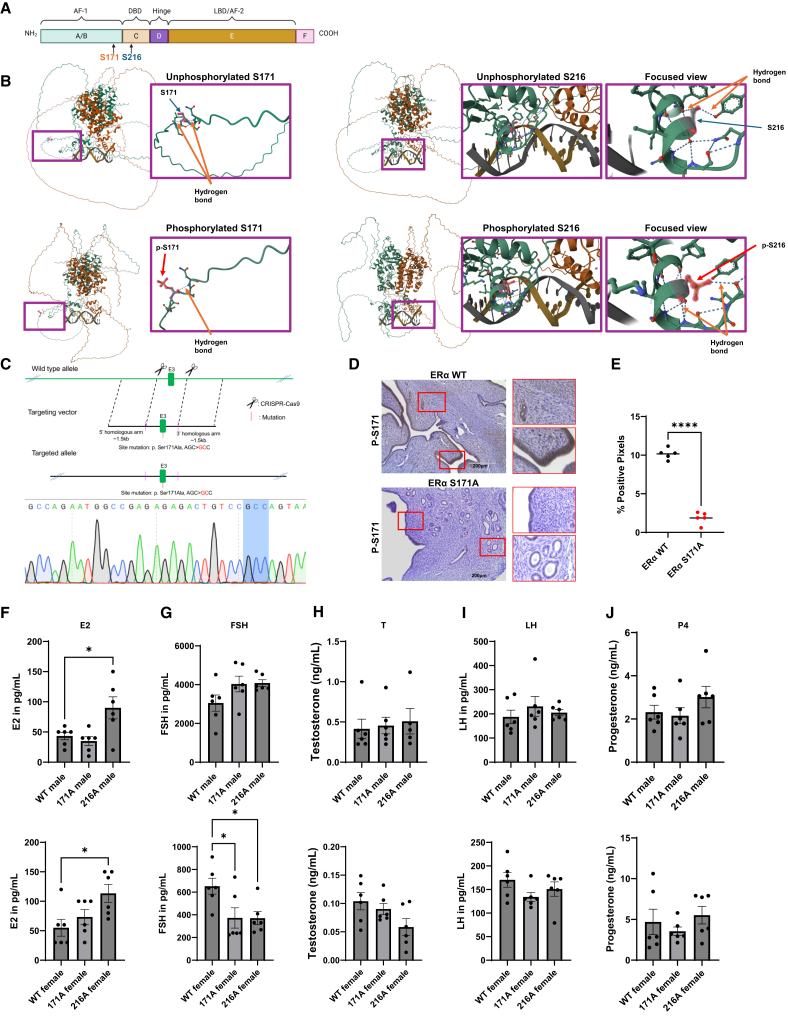
Generation of S171A mice and plasma hormone levels in S171A and S216A mice (A) Schematic representation of the mouse estrogen receptor alpha (ERα), including domains A through F: A/B, Activation Function-1 (AF-1); C, DNA-binding domain (DBD); D, hinge domain; E, ligand-binding domain (LBD) and Activation Function-2 (AF-2); F, carboxyl-terminal domain. Phosphorylation sites S171 (orange) in the AF-1 domain and S216 (blue) in the DBD are indicated by arrows. (B) Predicted 3D structures of ERα generated by AlphaFold3 highlighting changes between the unphosphorylated (top) and phosphorylated (bottom) states. Overall predicted ERα structures with magnified views of the regions surrounding phosphorylation sites S171 and S216. Key molecular features are highlighted including hydrogen bonds (orange arrows), unphosphorylated serine residues (green arrows), and phosphoryl groups introduced upon phosphorylation (red arrows). This figure was generated using the Mol∗ 3D Viewer.^19^ (C) Targeting strategy for introducing a site-specific CRISPR-Cas9 knock-in mutation and confirmation by Sanger sequencing. Top: Schematic representation of the wild-type allele, targeting vector, and successfully targeted allele. The CRISPR-Cas9 system introduces a mutation at exon 3 (E3) replacing the serine codon (AGC) with an alanine codon (GCC) to generate the Ser171Ala (S171A) mutation. Bottom: Sanger sequencing results from DNA isolated from the S171A mouse tail confirm the successful introduction of the mutation. The targeted site is highlighted in blue showing the codon replacement from AGC (serine) to GCC (alanine). (D) Immunohistochemical detection of phosphorylated ERα at serine 171 (P-S171) in uterine cross sections from adult female WT (top) and S171A mutant (bottom) mice (*n* = 5 per genotype). Sections were incubated with a phospho-specific antibody targeting human pSer167. Brown DAB staining denotes P-S171-positive area. Scale bars, 200 μm. Quantification of P-S171-positive area was conducted using pixel-based positive-pixel detection in QuPath across five randomly selected fields per animal. (E) Quantification of P-S171-positive pixels using QuPath software. Each dot represents a randomly selected field. Data are expressed as mean ± SEM. Statistical significance was determined using an unpaired t test (∗∗∗∗*p* < 0.0001). (F–J) Plasma hormone concentrations were measured in 3-month-old mice of different genotypes (WT, S171A, and S216A) and sexes. Panels show levels of (F) estradiol (E2, pg/mL), (G) follicle-stimulating hormone (FSH, pg/mL), (H) testosterone (T, ng/mL), (I) luteinizing hormone (LH, pg/mL), and (J) progesterone (P4, ng/mL). Data are shown as individual points with the mean, with sample sizes of *n* = 6 mice per group. Statistical significance was determined using one-way ANOVA. Significance levels are indicated by asterisks: ∗*p* < 0.05.

Estrogen receptor alpha (ERα) is a nuclear hormone receptor that mediates the diverse physiological effects of estrogens in males and females, including regulation of reproductive functions, body composition, bone density, and metabolism.^1^^,^^2^^,^^3^^,^^4^ ERα functions as a ligand-activated transcription factor modulating gene expression upon estrogen binding.^5^^,^^6^ Beyond ligand binding, ERα activity is influenced by post-translational modifications, among which phosphorylation plays a critical role in modulating transcriptional activity, protein stability, DNA binding, and co-regulator interactions.^7^^,^^8^^,^^9^ ERα can also be activated independently of ligand through phosphorylation at residues such as S118 and S167 by growth-factor-regulated kinases, including MAPK and PI3K/AKT. S118 is primarily mediated by the MAPK/ERK pathway in response to extracellular signals such as epidermal growth factor (EGF).^10^^,^^11^^,^^12^^,^^13^ Phosphorylation at S167, regulated by the PI3K/AKT and 90-kDa ribosomal S6 kinase (pp90rsk1) signaling cascade, can be activated by insulin-related growth factor I and EGF, further supporting ligand-independent ERα activity and contributing to its transcriptional and non-genomic functions.^14^^,^^15^ Phosphorylation occurs on several serine residues within the functional domains of ERα, leading to conformational changes that affect receptor activity and subsequent signaling pathways. The functional domains of ERα include the transcriptional activation function-1 (AF-1) domain, DNA-binding domain (DBD), a hinge domain, ligand-binding domain (LBD), activation function-2 (AF-2) domain, and carboxyl-terminal F domain.^8^^,^^16^^,^^17^^,^^18^ A schematic representation of the domain structure is provided in Figure 1A.

The physiological importance of ERα has been demonstrated in mouse models through the deletion of ERα protein and ERα domain-specific deletions.^1^^,^^2^^,^^20^ Global ERα knockout (ERKO) mice exhibited infertility in both male and female mice, demonstrating a central role for ERα in reproductive function.^21^^,^^22^Domain-specific knockouts, such as those targeting the DNA-binding domain (EAAE and NERKI)^23^^,^^24^ and AF-1 domain,^25^ similarly disrupted fertility. ERα also regulates adipose tissue distribution and metabolism, as evidenced by increased adipose tissue weight and adipocyte size in ERKO mice. Mice lacking nuclear ERα signaling (H2NES) also exhibited increased adiposity.^26^ ERα regulates bone density and remodeling through both genomic and membrane-initiated pathways, with effects that are sex-, compartment-, and cell-type-specific. Whole-body ERKO models display complex skeletal phenotypes due in part to elevated circulating sex steroids.^27^ The AF-1 domain is essential for estrogen-mediated protection of trabecular bone but is dispensable for cortical bone.^28^ In contrast, cortical bone homeostasis is partly dependent on membrane-associated ERα signaling.^29^

Recent studies have demonstrated the importance of several ERα phosphorylation sites in regulating physiological processes and body composition in mice and in modulating transcription. Using mixed genetic background C57B6:129 mice, Negishi et al. found that phosphorylation of ERα at serine 216 (S216) played a crucial role in microglial-mediated neuroinflammation. S216A mutant mice exhibited elevated microglial activation, increased expression of pro-inflammatory cytokines, and impaired motor coordination, indicating an anti-inflammatory function for S216 phosphorylation in the brain.^30^^,^^31^^,^^32^ The serine 212 (S212) phosphorylation site in humans, corresponding to murine S216 in human ERα, was demonstrated to be critical for DNA binding and estrogen-regulated gene expression *in vitro.*^33^ Loss of phosphorylation at serine 122 (S122A mutation) in C57BL6 female mice resulted in increased fat mass and larger adipocyte size, whereas males exhibited reduced cortical bone response to estradiol (E2).^30^^,^^31^ The serine 118 (S118) phosphorylation site in humans corresponding to murine serine 122 has been demonstrated to play a critical role in modulating ERα-regulated gene expression, influencing breast cancer prognosis and contributing to endocrine therapy resistance.^34^^,^^35^^,^^36^^,^^37^ Previous studies from this laboratory have demonstrated that phosphorylation at serine 167 (S167), located in the AF-1 domain of human ERα, enhanced ERα interaction with ERα-target gene promoters to regulate gene transcription *in vitro.*^37^^,^^38^

A recent study on the human S118 phosphorylation site demonstrated that phosphorylation induced significant conformational changes in ERα by disrupting critical hydrophobic interactions,^39^ emphasizing the role of phosphate groups in driving structural transitions. The murine phosphorylation sites corresponding to human S167 and S212 (S171 and S216, respectively) are predicted to affect ERα conformation. S171 is located in the AF-1 domain, whereas S216 is found in a highly conserved region of the DNA-binding domain common to the nuclear receptor superfamily (Figure 1A). Because S216 is situated between the two zinc fingers that interact with the major groove of DNA, phosphorylation at S216 would be expected to influence ERα DNA binding and ERα-regulated gene expression significantly.

To investigate the impact of the ERα phosphorylation at S171 and S216 on mouse physiology, two knock-in (KI) C57B6 mouse models carrying serine-to-alanine mutations at S171 and S216 (S171A and S216A^32^) were generated. These models mimic non-phosphorylatable forms of ERα at two sites critical for ERα-regulated gene expression and permit the study of phosphorylation-specific effects on estrogen signaling. Physiological analyses of these models identified distinct site-specific and sex-specific effects on hormone levels, body composition, fertility, uterine responses to E2, tissue-specific ERα expression, and plasma metabolites. These findings provide new insights into how site-specific phosphorylation of ERα impacts a myriad of physiological processes with implications for health and disease.

## Results

### Predicted structural effects of ERα phosphorylation at S171 and S216

To investigate the structural impact of phosphorylation at S171 and S216, AlphaFold^40^ was used to predict and compare the three-dimensional structures of phosphorylated and unphosphorylated ERα at these two sites. AlphaFold predicted the three-dimensional ERα dimer structure bound to DNA in the absence or presence of phosphorylation at sites S171 or S216 (Figure 1B). In the absence of phosphorylation, S171 forms hydrogen bonds with asparagine (ASN) 173 and leucine (LEU) 169. Phosphorylated S171 retains the hydrogen bond with ASN173 but loses the hydrogen bond with LEU169. Unphosphorylated S216 forms hydrogen bonds with phenylalanine (PHE) 212 and tyrosine (TYR) 223. Phosphorylated S216 loses these hydrogen bonds with PHE212 and TYR223 and instead forms hydrogen bonds with glycine (GLY) 219 and ASN221. These predicted structural alterations in the 3-D conformation of ERα induced by phosphorylation at sites S171 and S212 would likely have a marked impact on ERα interaction with DNA and thus significantly impact ERα-regulated gene expression. To assess whether S to A mutations alter ERα conformation in a manner similar to the unphosphorylated state, AlphaFold3 structural modeling was performed. The analysis demonstrated that the S171A and S216A mutants resemble key hydrogen-bonding interactions of the unphosphorylated ERα (Figure S1A).

### Generation of phospho-KI mice

Serine 171 in mice corresponds to serine 167 in the human ERα (Figure S1B). To generate the S171A mouse model, a serine-to-alanine mutation was introduced at amino acid residue 171 within exon 3 of the mouse ESR1 gene using the CRISPR-Cas9 system in C57BL6 mice by Biocytogen (Waltham, MA). The mutation was designed to replace the serine codon (AGC) with an alanine (GCC). A targeting vector was constructed to include this mutation and 1.5 kb homologous arms on each side of the target site to initiate the homologous recombination (Figure 1C, top). Several sgRNAs were designed and tested by the Universal CRISPR Activity Assay for efficacy and specificity (Figure S1C). The most active sgRNAs, sgRNA2 and sgRNA14, were selected to direct Cas9 to the specific ESR1 sequence and initiate genome editing (Figure S1D). Sequencing of purified genomic DNA extracted from S171A mouse tail samples confirmed the replacement of AGC with GCC at the target site (Figure 1C, bottom panel). To determine whether the serine-to-alanine mutation inhibited ERα phosphorylation at amino acid 171, immunohistochemistry (IHC) was performed on uterine tissue known to exhibit high expression of ERα from adult (3-month-old) S171A female mice. Phosphorylation was detected using a phospho-specific antibody targeting the phosphorylation of human serine 167, which is homologous to mouse serine 171. IHC analysis comparing uterine cross sections from wild-type (WT) or S171A mice incubated with anti-p-S167 antibody demonstrated loss of immunoreactivity in the S171A uterus (Figures 1D and 1E). Dr. Masahiko Negishi kindly provided pre-validated ERα S216A KI mice backcrossed four times to C57BL6 to yield S216A KI mice in a pure C57BL6 background. The S171A and S216A KI models were compared with WT C57BL6 mice to assess the physiological consequences of point mutations in ERα phosphorylation sites.

### Site-specific and sex-specific effects of ERα phosphorylation on plasma hormone levels

Plasma hormone levels were measured in three-month-old male and female mice (staged at diestrus) (Figures 1F–1J). In male mice, E2 levels were significantly elevated in S216A mice compared with WT (90.00 pg/mL vs. 43.33 pg/mL, *p* = 0.0167), whereas no significant change was observed in S171A males. No significant differences were found in follicle-stimulating hormone (FSH), testosterone (T), luteinizing hormone (LH), or progesterone (P4) levels among the male groups. In female mice, E2 levels were significantly higher in S216A mice compared with WT (113.30 pg/mL vs. 55.00 pg/mL, *p* = 0.0035), while S171A females did not show a significant change. FSH levels were significantly reduced in both S171A (372.8 pg/mL vs. 652.2 pg/mL, *p* = 0.043) and S216A (370.2 pg/mL vs. 652.2 pg/mL, *p* = 0.041) females relative to WT. Similar to the findings in males, no significant differences in P4, LH, or T levels were detected in S171A or S216A females compared with WT. These distinct, sex- and mutation-specific alterations in plasma E2 and FSH concentrations underscore a critical role for ERα phosphorylation in regulating hypothalamic-pituitary-gonadal (HPG) axis function providing a context for investigating how site-specific ERα phosphorylation modulates systemic physiology, including body composition, fertility, and tissue-specific estrogen responsiveness.

### Change in ERα protein levels across various tissues

**Figure 2:**
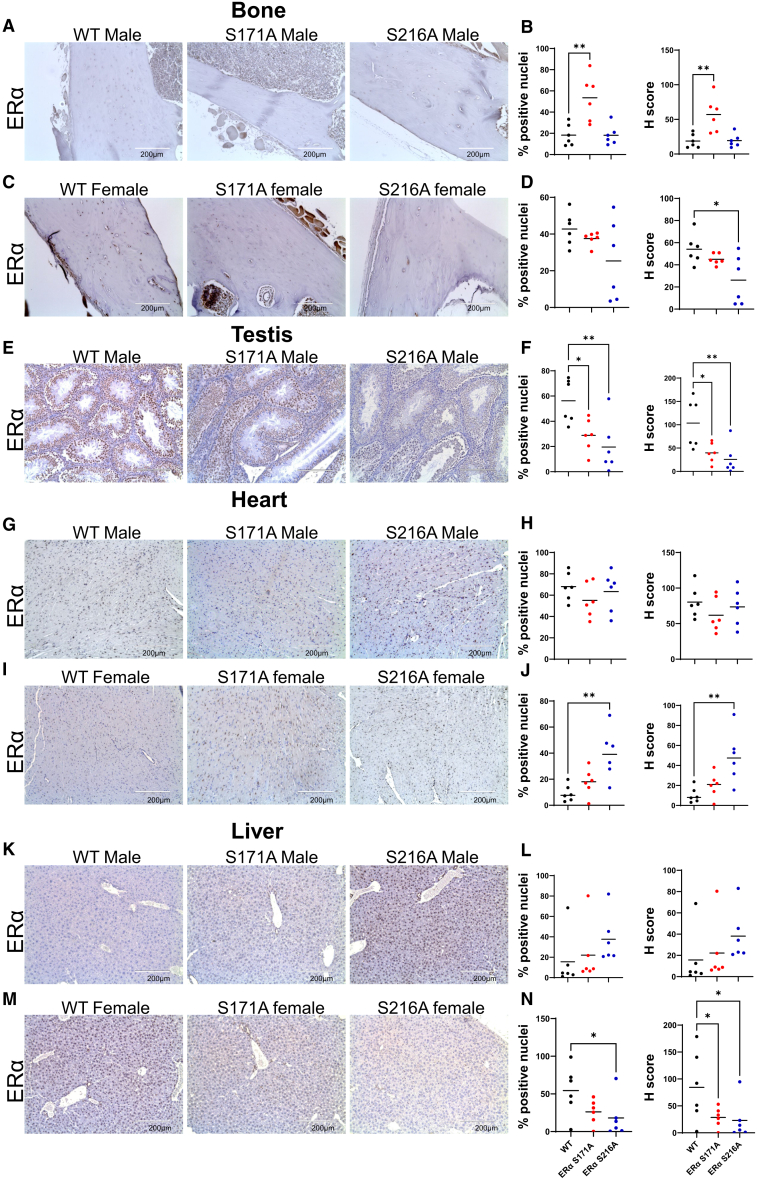
Total ERα protein levels in various tissues in WT, S171A, and S216A mice Representative IHC images (A, C, E, G, I, K, and M) are shown for male and female mice in various tissues with corresponding quantitative assessments of ERα protein expression (B, D, F, H, J, L, and N). Panels illustrate ERα staining in bone, testis, heart, and liver. Quantitative data in scatterplots show the percentage of ERα-positive cells and H-score for each tissue type. Scale bars represent 200 μm. Data are presented as individual data points with the mean. *n* = 6 mice per group. Statistical analysis was performed using one-way ANOVA. Significance levels are indicated by asterisks: ∗*p* < 0.05, ∗∗*p* < 0.01.

To determine if mutations at these two phosphorylation sites impact total ERα protein expression levels across multiple mouse tissues, IHC to measure ERα was conducted on 3-month-old mice (*n* = 6 per group) for both sexes with female mice staged at diestrus. ERα localization remained predominantly nuclear in all tissues. Protein expression levels were quantified by both the percentage of ERα-positive cells and staining intensity (H-score), revealing distinct, site- and sex-specific alterations (Table 1). In bone (Figures 2A–2D), S171A increased ERα in males (Figures 2A and 2B) with no change in S216A, whereas in females (Figures 2C and 2D), S216A reduced ERα with no change in S171A. In testis (Figures 2E and 2F), ERα was decreased in both S171A and S216A males. In heart (Figures 2G–2J), no significant changes were detected in males (Figures 2G and 2H), whereas in females (Figures 2I and 2J), S216A increased ERα with no change in S171A. In liver (Figures 2K–2N), no significant changes were detected in males (Figures 2K and 2L), whereas in females (Figures 2M and 2N), ERα was reduced in both S171A and S216A. No site-specific differences were found in kidney (Figures S2A–S2D), lung (Figures S2E–S2H), brain (Figures S2I–S2L), mammary gland (Figures S2M and S2N), or uterus (Figures S2O and S2P). IgG negative control images (Figure S2Q) confirmed no detectable non-specific staining. These findings demonstrate that loss of ERα phosphorylation at S171 and S216 alters ERα protein levels in a tissue- and sex-specific manner without affecting its nuclear localization.

**Table 1:** Summary of ERα protein levels across various tissues

Tissue	S171A male	S171A female	S216A male	S216A female
Bone	Increased % positive and H-score	NS	NS	Decreased H-score
Mammary gland	NA	NS	NA	NS
Heart	NS	NS	NS	Increased % positive and H-score
Testis	Decreased % positive cells and H-score	NA	Decreased % positive cells and H-score	NA
Liver	NS	Decreased % H-score	NS	Decreased % positive cells and H-score
Kidney	NS	NS	NS	NS
Brain	NS	NS	NS	NS
Lung	NS	NS	NS	NS
Uterus	NA	NS	NA	NS

NS, non-significant; NA, not available.

### Site-specific and sex-specific impact of ERα phosphorylation on fertility

**Figure 3:**
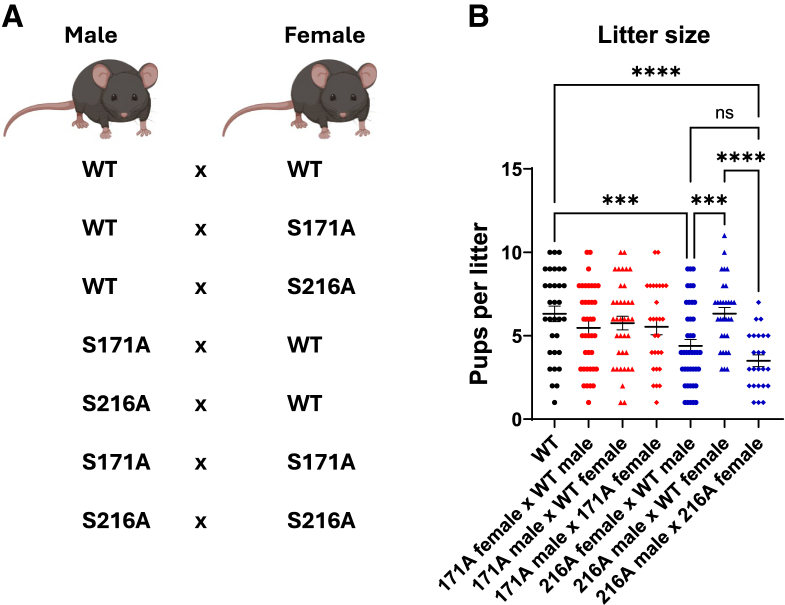
Fertility assessment of S171A and S216A mice (A) Schematic illustrating the mating pairs analyzed during the six-month fertility study. (B) Litter size (number of pups per litter) for each mating pair. Data are presented as individual data points with the mean. *n* = 6 mating pairs per group. Statistical analysis was performed using one-way ANOVA. Significance levels are indicated by asterisks: ∗∗∗*p* < 0.001, ∗∗∗∗*p* < 0.0001.

ERα expression in multiple tissues is essential for mouse fertility.^41^^,^^42^ Previous reports identified infertility in both male and female total ERKO mice.^21^^,^^22^^,^^43^^,^^44^^,^^45^ Infertility also occurred in tissue-specific, ERKOs, including uterine epithelial-specific ERKO mice that resulted in implantation failure,^46^ adipocyte and hypothalamic ERα female knockout mice that exhibited elevated serum E2,^47^ neuron-specific ERα female knockout mice that exhibited impaired LH secretion and GnRH neuron activation,^48^ and pituitary-specific female ERKO mice that exhibited a disrupted estrous cycle.^49^ A six-month fertility study was conducted to assess whether S171A or S216A mutations impacted reproductive outcomes. WT males and WT females (proven breeders) were paired with mutant mice, and mutant-to-mutant matings were also evaluated (Figure 3A). In males, the S171A and S216A mutations did not significantly affect fertility, as indicated by comparable time between litters, number of litters, and male-to-female pup ratios relative to WT (Figures 3B and S3A–S3C). In females, S216A mice exhibited a significant reduction in litter size. S216A females mated with WT males had fewer pups per litter compared with WT females (∼4.33 vs. ∼6 pups, *p* = 0.0004), and an even greater reduction was seen in S216A female × S216A male matings (∼2.9 pups/litter, *p* < 0.0001) (Figure 3B). Conversely, no fertility defects were observed in S171A females. These findings identify a phosphorylation-site-specific and sex-specific impairment in fertility, specifically associated with the S216A mutation in females.

### Impact of ERα phosphorylation on uterotrophic responses to estradiol

**Figure 4:**
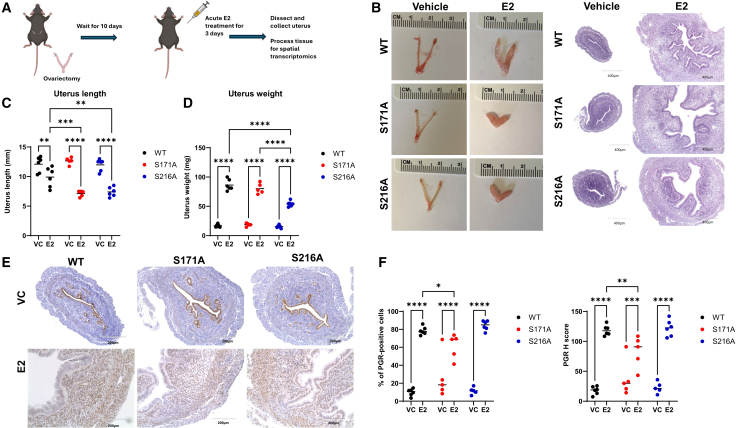
Uterotrophic response in ovariectomized mice following E2 treatment (A) Schematic representation of the uterotrophic response experiment. Following ovariectomy and a 10-day recovery period, mice were administered a subcutaneous dose of either vehicle or E2 (daily subcutaneous injection of 10 μg/kg E2 dissolved in sesame oil for 72 h). (B) Representative H&E-stained sections of mouse uterus cross sections (scale bar: 400 μm) in WT, S171A, and S216A mice. (C) Quantitative analysis of uterine length. (D) Quantitative analysis of uterine weight. (E) Representative immunohistochemical images of immunostaining with the anti-PGR antibody in uterine cross sections of WT and mutant mice (scale bar: 200 μm). (F) Quantification of the percentage of PGR-immunopositive cells (left) and corresponding H-scores (right) (scale bar: 200 μm). Data are presented as individual data points with the mean. n = 5–6 mice per group. Statistical analysis was performed using two-way ANOVA followed by Tukey post hoc test. Significance levels are indicated as follows: ∗*p* < 0.05, ∗∗*p* < 0.01, ∗∗∗*p* < 0.001, ∗∗∗∗*p* < 0.0001.

To further evaluate the impact of ERα phosphorylation on female mice, uterotrophic responses to exogenous E2 were assessed in ovariectomized female mice. Following ovariectomy and a subsequent acute, 72-h subcutaneous administration of E2 (Figure 4A), significant morphological, physiological, and ERα target gene alterations were observed in the uteri of both S171A and S216A mice compared with WT mice. Although no significant differences in uterine morphology and histology were observed in mice dosed with the vehicle, distinct morphological differences were evident in both mutant mouse models dosed with E2 (Figure 4B). Specifically, both S171A and S216A mutants exhibited shorter uterine lengths compared with WT (7.12 mm vs. 9.91 mm, *p* < 0.0001; 7.41 mm vs. 9.91 mm, *p* = 0.0001, respectively) (Figures 4B and 4C). E2-dosed S216A mice also displayed significantly reduced uterine weight compared with WT (54.23 mg vs. 86.37 mg, *p* < 0.0001) (Figure 4D). Additionally, no significant change was found in total abdominal fat weight compared with WT (Figure 4A). These findings indicate that ERα mutations at the S171 and S216 sites modulate uterine morphology and uterine weight gain in response to E2. Immunohistochemical measurement of Ki67, an E2-induced cell proliferation marker, and progesterone receptor (PGR), an established marker of ERα activation, was performed in uteri from both vehicle- and E2-dosed groups (Figures S4B, S4C, 4E, and 4F). Although E2 induced increased cell proliferation in WT, S171A, and S216A uteri, the Ki67 expression level was significantly lower in both mutant genotypes compared with WT, indicating a reduced proliferative response to E2 in the S171A and S216A mutants (Figures S4B and S4C). PGR was detected predominantly in uterine luminal and glandular epithelial cells in vehicle-dosed animals. E2 increased both the number of PGR-positive cells and the PGR staining intensity (quantified by H-score) as well as stromal PGR expression in all mice (Figures 4E and 4F). However, a phosphorylation-site-specific alteration in PGR response was noted among S171A and S216A mice compared with WT mice. E2-dosed S171A mice exhibited a reduction in both PGR-positive cell number and staining intensity, whereas S216A mice showed no significant change in either the number of PGR-positive cells or overall PGR intensity. The complete two-way ANOVA and Tukey post hoc results are provided in Table S1. These results demonstrate that site-specific ERα phosphorylation altered estrogen signaling responses for both Ki67 and PGR expression in the uterus.

### Spatial transcriptomics revealed ERα phosphorylation-dependent, cell-type-specific uterine responses to E2

**Figure 5:**
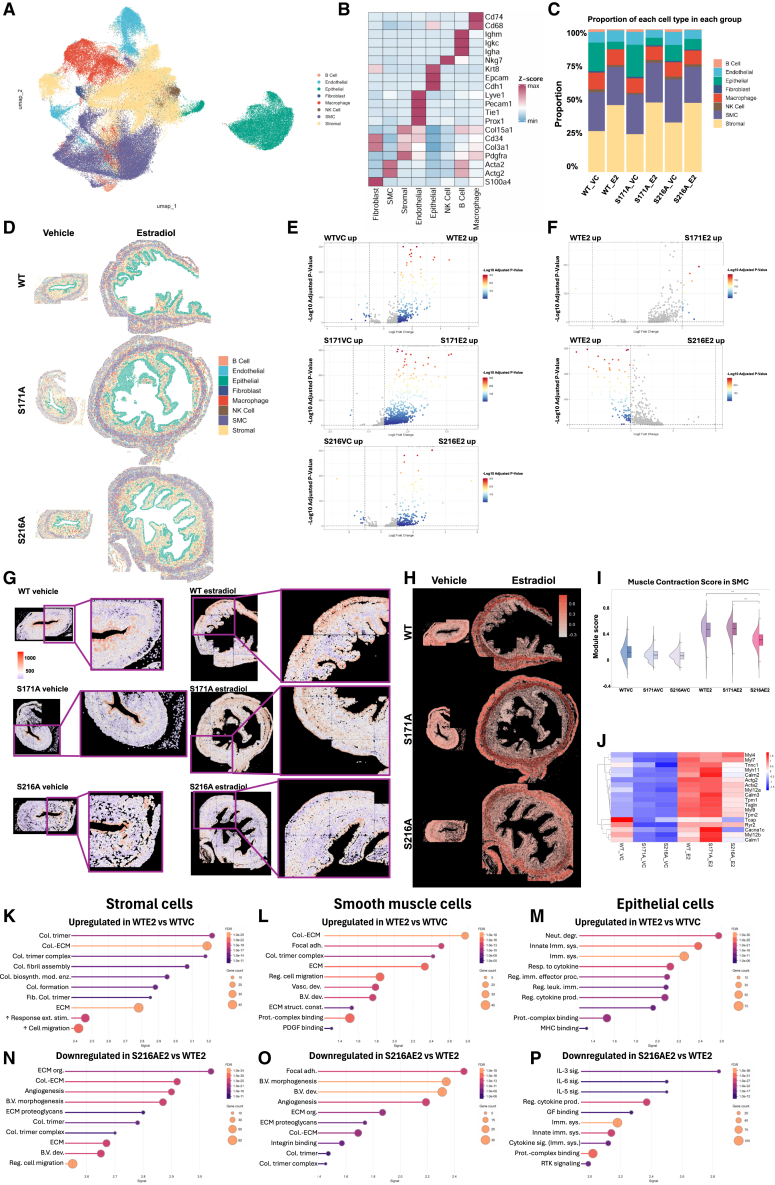
Spatial transcriptomic and functional analysis of ERα phosphorylation-dependent 17β-estradiol (E2) responses in the uterus (A) UMAP projection of uterine cell clusters from NanoString CosMx spatial transcriptomics (1,000-plex gene panel) across genotypes (WT, S171A, S216A) and treatments (vehicle, E2). (B) Cell type annotation using marker genes: fibroblasts (S100a4), smooth muscle cells (SMC; Acta2, Actg2), stromal cells (Pdgfra, Col3a1), endothelial cells (Pecam1, Prox1), epithelial cells (Cdh1, Epcam), NK cells (Nkg7), B cells (Ighm), and macrophages (Cd68). (C) Quantification of cell populations per genotype and treatment. (D) Spatial distribution of cell types in uterine cross sections. (E and F) Volcano plots of differential gene expression. Genes with significant expression changes (adjusted *p* < 0.05, log2 fold change > ±1) are highlighted in colored dots, with the scale indicating the log10 adjusted *p*-value. Non-significant genes are represented by gray dots. (G) Spatial mapping of transcript counts, with a magnified view emphasizing the myometrium. (H) Spatial visualization of the muscle contraction module scoring across all uterine cells. (I) Muscle contraction module score in smooth muscle cell (SMC). Statistical test: two-sided Wilcoxon rank-sum tests, ∗∗∗ (*p* < 0.001). (J) Heatmap of genes used for muscle contraction module scoring in SMC. (K-M) Pathway enrichment analysis of genes upregulated in WT_E2 vs. WT_VC: (K) stromal cells, (L) SMC, (M) Epithelial cells. (N–P) Pathway enrichment analysis of genes downregulated in S216A_E2 vs. WT_E2: (N) stromal cells, (O) SMC, (P) Epithelial cells. The *x* axis represents enrichment signal values, and the size of each dot corresponds to the number of genes in the enriched category. The color scale indicates the false discovery rate. The full names of the abbreviated pathways are provided in Table S4.

To further understand how ER phosphorylation modulates E2-regulated transcription in the uterus, spatial transcriptomics analysis was utilized to capture cell-type-specific transcriptional changes in uterine tissues at single-cell resolution. The NanoString CosMx platform, designed for spatial transcriptomics at single-cell resolution using a 1000-plex gene panel, was employed to analyze the cross sections of mouse uteri from the uterotrophic assay (Figure 5A). Cells with fewer than 10 detected genes or genes expressed in fewer than 3 cells were filtered out. Quality control metrics were assessed to ensure data integrity (Figures S5B and S5C). Cell segmentation was performed using a machine-learning-based algorithm by NanoString (Figure S5D), which identified cell populations across experimental conditions: WT_VC (9,436 cells), S171A_VC (10,962 cells), S216A_VC (7,154 cells), WT_E2 (18,763 cells), S171A_E2 (37,474 cells), and S216A_E2 (42,647 cells). Uniform Manifold Approximation and Projection (UMAP) analysis demonstrated the clustering of cells into distinct subpopulations (Figure 5A), with identities validated through established marker expression profiles.^50^^,^^51^ This demonstrated the heterogeneity of cell types in the mouse uterus. The identified clusters include fibroblasts (S100a4), smooth muscle cells (SMCs) (Acta2, Actg2), stromal cells (Pdgfra, Col3a1, Cd34, Col15a1), endothelial cells (Prox1, Tie1, Lyve1, Pecam1), epithelial cells (Cdh1, Epcam, Krt8), natural killer (NK) cells (Nkg7), B cells (Ighm, Igkc, Igha), and macrophages (Cd68, Cd74) (Figure 5B). Each cell type was counted, revealing stromal cell and epithelial cell proliferation following acute E2 treatment across WT, S171A, and S216A genotypes (Figure 5C). The spatial distributions of all cell types were visualized in Figure 5D.

Differential gene expression analysis across cell types identified significant transcriptional changes following acute E2 exposure. E2 administration to mice induced upregulation of genes across all three genotypes, consistent with the established role of ERα as a transcriptional factor in uterine tissues (Figure 5E). The S216A genotype displayed distinct transcriptional profiles compared with WT, characterized by extensive downregulation of gene expression (Figure 5F). A comprehensive analysis of gene expression profiles across cell types and genotypes is provided in Figure S6. Spatial mapping revealed that the downregulated transcripts in S216A mice were predominantly localized to the myometrium, where the overall signal intensity was markedly reduced (Figure 5G), emphasizing the advantage of spatial transcriptomics in exposing the anatomical localization of altered gene expression patterns.

To investigate the functional implications of E2-induced transcriptional changes in the myometrium, muscle contraction-associated genes were analyzed using the Seurat AddModuleScore tool. Module scoring across all uterine cells revealed a significant reduction in muscle contraction module activity in the S216A compared with WT (Figure 5H). Further analysis focused explicitly on SMCs within the myometrium, which demonstrated markedly reduced contraction module activity in S216A relative to WT (Figure 5I). The contraction module included genes essential for contractile function,^52^^,^^53^ such as actin-binding proteins (Acta2, Actg2, Tagln), myosin heavy and light chains (Myh11, Myl4, Myl7, Myl9, Myl12a, Myl12b), tropomyosin and calcium-regulatory proteins (Tpm1, Tpm2, Tnnc1, Tcap, Calm1-3), and calcium channels (Cacna1c, Ryr2). A heatmap demonstrated the downregulation of these genes in S216A SMCs (Figure 5J). This suppression corresponds with the transcriptional decline observed in the S216A myometrium (Figure 5G) and suggests impaired smooth muscle function, potentially compromising uterine contractility in response to E2.

To further investigate the impact of E2 on uterine biology, pathway enrichment analysis of differentially expressed genes was performed across stromal, smooth muscle, and epithelial cell populations. Acute E2 treatment in WT mice robustly activated pathways critical for preparation for pregnancy/parturition, including extracellular matrix remodeling (e.g., collagen trimer assembly; Figure 5K), angiogenesis and focal adhesion (Figure 5L), and immune modulation (cytokine signaling, MHC protein binding; Figure 5M) in stromal cells, SMCs, and epithelial cells, respectively. These processes collectively facilitate uterine remodeling, vascular support, and immune regulation, which is necessary for embryo implantation and uterine receptivity. In contrast, the S216A uterus exhibited broad impairment in E2-driven pathway activation. Stromal cells exhibited deficient collagen reorganization and angiogenesis compared with WT (Figure 5N), suggesting compromised ECM integrity and vascularization. SMCs displayed attenuated focal adhesion signaling and ECM organization (Figure 5O), corresponding with reduced contractile gene expression in these cells that is critical for coordinated contractions during parturition (Figure 5J). Epithelial cells, which mediate critical embryo-uterine interactions, exhibited downregulation of cytokine signaling (e.g., IL-6, IL-3, IL-5) and growth factor responses (Figure 5P). These gene expression deficiencies likely disrupt the communication required for embryo attachment. The complete results of pathway enrichment analysis, including Gene Ontology, KEGG, WikiPathways, and Reactome, are available in Figures S7–S12. These cell-type-specific deficits likely contribute to the S216A subfertility phenotype by impairing the sophisticated uterine remodeling required for implantation and pregnancy establishment, as well as impairing the coordinated waves of myometrial contraction necessary for parturition. Taken together, these results imply the importance of ERα phosphorylation at S216 in E2-induced transcriptional programs across uterine compartments.

### Site- and sex-specific changes in plasma metabolites

**Figure 6:**
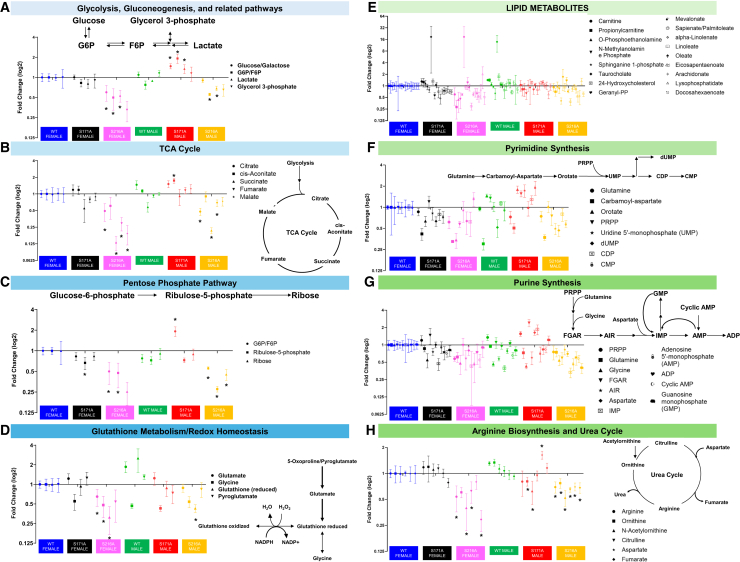
Metabolomic profiling of plasma metabolites in 3-month-old WT, S171A, and S216A mice (A) Changes in metabolites associated with glycolysis, gluconeogenesis, and related pathways. Key metabolites include glucose/galactose, glucose-6-phosphate/fructose-6-phosphate (G6P/F6P), lactate, and glycerol-3-phosphate. (B) Changes in tricarboxylic acid (TCA) cycle metabolites including citrate, *cis*-aconitate, succinate, fumarate, and malate, with a schematic representation of the TCA cycle. (C) Changes in pentose phosphate pathway metabolites including G6P/F6P, ribulose-5-phosphate, and ribose. (D) Changes in metabolites related to glutathione metabolism and redox homeostasis, such as glutamate, glycine, reduced glutathione, and pyroglutamate, with a schematic illustration of the glutathione redox cycle. (E) Changes in lipid metabolites, including free fatty acids, glycerolipids, and phospholipids, with a schematic representation of key lipid metabolic pathways. (F) Changes in metabolites associated with pyrimidine synthesis, such as orotate, uridine monophosphate (UMP), cytidine monophosphate (CMP), and deoxythymidine monophosphate (dTMP), with a schematic illustration of the pyrimidine synthesis pathway. (G) Changes in metabolites involved in purine synthesis, including inosine monophosphate (IMP), guanosine monophosphate (GMP), adenosine monophosphate (AMP), and their triphosphate forms, with a schematic representation of the purine synthesis pathway. (H) Changes in metabolites related to arginine biosynthesis and the urea cycle, such as citrulline, ornithine, arginine, and urea, with a schematic depiction of the urea cycle. The data are presented as mean ± SEM (*n* = 6 mice per group) and plotted as log2-fold changes relative to WT females. Metabolite levels were compared using two-tailed unpaired Student t tests, with *p* < 0.05 considered statistically significant. Asterisks denote values significantly different from WT controls (*p* < 0.05).

ERα is a key regulator of metabolic processes that impact energy balance, glucose homeostasis, and lipid metabolism.^2^^,^^3^ Given the crucial role of ERα in these pathways and the marked alterations in hepatic ERα protein levels in S171A and S216A mutant mice (Figures 2E and 2H), a plasma metabolomic analysis was conducted to determine whether tissue-specific transcriptional alterations translate into systemic metabolic effects in a site- and sex-dependent manner. Untargeted plasma metabolomics was performed in 3-month-old phospho-mutant and wild-type mice of both sexes. This unbiased profiling of circulating metabolites across carbohydrate, lipid, and amino acid pathways served as a functional readout of how ERα phosphorylation status influences whole-body metabolic homeostasis. Metabolomic analysis of plasma from 3-month-old WT, S171A, and S216A mice with female mice staged at diestrus revealed distinct site- and sex-specific metabolic alterations across multiple pathways. The S216A mutation resulted in a generally suppressed metabolic profile, with significant reductions in metabolites involved in carbohydrate metabolism (Figures 6A–6C), antioxidant capacity and redox homeostasis (Figure 6D), lipid metabolism (Figure 6E), nucleotide metabolism (Figures 6F and 6G), and amino acid metabolism (Figure 6H), affecting both males and females. Notably, glycolysis was significantly repressed in S216A mice as evidenced by significantly lower lactate levels, which is the end product of glycolytic fermentation, compared with WT controls (Figure 6A). This repression was more pronounced in females, with reductions observed in all measured glycolytic metabolites. The tricarboxylic acid (TCA) cycle was also reduced in both S216A males and females, as indicated by decreased levels of TCA intermediates (Figure 6B). Similar to glycolysis, the decrease in TCA cycle metabolites was more pronounced in females than males.

In contrast to S216A, the S171A mutation demonstrated more variable, sex-dependent plasma metabolite alterations. In S171A males, glycolytic activity appeared upregulated with elevated glucose and lactate levels compared with WT controls (Figure 6A), suggesting enhanced energy metabolism. However, this increased glycolytic response was absent in S171A females (Figure 6A), indicating a sex-specific metabolic response to the S171A mutation. Further examination of related metabolic pathways revealed phosphorylation-site-specific effects, particularly in the pentose phosphate pathway (PPP), a key branch of the glycolysis pathway that provides precursors for nucleotide synthesis. Both male and female S216A mice exhibited decreased levels of PPP products (Figure 6C) as compared with the WT group, including ribulose-5-phosphate and ribose, with males showing significant reductions (*p*-value <0.05) in both these compounds and females showing a significant reduction in ribulose-5-phosphate. This decrease in PPP activity corresponded with lower levels of purine and pyrimidine nucleotide metabolites in S216A males, while females displayed relatively unchanged nucleotide levels (Table S2), indicating a male-specific reduction in nucleotide synthesis capacity.

Given the observed altered metabolism of nucleotides, the building blocks of DNA and RNA, the subsequent examination focused on dysregulation of amino acids, which are the building blocks of peptides and proteins. Specifically, the redox homeostasis pathway (Figure 6D) was impaired by the S216A mutation, with both male and female S216A mice showing significantly reduced levels of reduced glutathione (GSH), a critical antioxidant, along with its precursors, glutamate, and glycine. This reduction suggested a compromised antioxidant defense in the S216A group, likely due to diminished GSH synthesis rather than increased consumption, as oxidized glutathione (the conversion product of reduced glutathione) remained unchanged across groups. Overall, these findings identified a profound impact of the S216A mutation on plasma metabolites consistent with a widespread suppression of energy, nucleotide, and redox metabolism. In contrast, the S171A mutation exhibited a more variable, sex-specific effect on plasma metabolites, particularly characterized by enhanced glycolysis in males. The site- and sex-dependent alterations in circulating glycolytic and lipid intermediates indicated a sustained reprogramming of energy utilization and storage in the KI lines, a phenotype expected to manifest at the whole-body level.

### Site-specific and sex-specific effects of ERα phosphorylation on body composition

**Figure 7:**
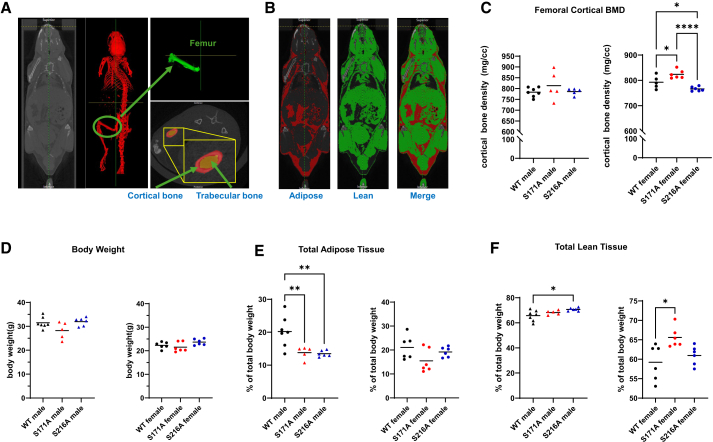
Body composition analysis of S171A and S216A mice (A) Representative micro-CT images displaying segmentation of the femoral bone in coronal view. Insets highlight separate segmentation of cortical and trabecular bone in axial view. (B) Micro-CT images illustrating segmentation of adipose tissue (red), lean tissue (green), and a merged image in coronal view. (C) Quantification of femoral cortical BMD (mg HA/cc) in WT, S171A, and S216A male and female mice. (D) Body weight of the mice used for Micro-CT analysis. (E) Total adipose tissue presented as a percentage of total body weight. (F) Total lean tissue presented as a percentage of total body weight. Data are shown as individual points with the mean, with sample sizes of n = 5–7 mice per group. Statistical significance was determined using one-way ANOVA. Significance levels are indicated by asterisks: ∗*p* < 0.05, ∗∗*p* < 0.01, ∗∗∗∗*p* < 0.0001.

To determine whether metabolomic alterations correspond to measurable physiological changes, body composition analysis was performed on three-month-old mice using micro-computed tomography (CT) to measure bone mineral density (BMD), adipose tissue, and lean tissue.^54^ This analysis evaluated whether ERα phosphorylation status drives site-specific differences in adiposity or lean tissue development, thereby linking molecular alterations to systemic phenotypes. Segmenting the cortical and trabecular bone of the femur and the separation of adipose and lean tissues are illustrated in Figures 7A and 7B, respectively. In male mice, no significant differences in femoral cortical BMD or cortical thickness were observed in either S171A or S216A mutants compared with WT (Figures 7C and S13). However, while no significant difference in overall body weight was observed (Figure 7D), both S171A and S216A males showed a significant reduction in adipose tissue percentage relative to WT (13.81% vs. 20.21%, *p* = 0.0064 for S171A; 13.49% vs. 20.21%, *p* = 0.0029 for S216A; Figure 7E). Additionally, S216A males exhibited a significant increase in lean mass compared with WT (70.73% vs. 65.78%, *p* = 0.0085; Figure 7F). In female mice, femoral cortical BMD was significantly increased in S171A mice (823.7 mg/cc vs. 792.7 mg/cc, *p* = 0.0372) but decreased in S216A mice (766.3 mg/cc vs. 792.7 mg/cc, *p* = 0.0393) relative to WT, without a change in cortical thickness (Figures 7C and S13). No significant differences in adipose tissue were detected in either S171A or S216A females (Figure 7E). However, S171A females showed a significant increase in lean mass compared with WT (65.61% vs. 59.22%, *p* = 0.0014; Figure 7F). These findings demonstrate that ERα phosphorylation has site- and sex-specific effects on body composition. Point mutations at S171 and S216 significantly reduced adipose tissue in males but not females. In contrast, effects on BMD were observed exclusively in females, with increased femoral cortical BMD in S171A mice and decreased BMD in S216A mice. These distinct patterns highlight the differential physiological roles of specific ERα phosphorylation sites in regulating adipose and bone.

## Discussion

The present study establishes new levels of complexity for ERα regulation of physiological processes via site-specific phosphorylation. By investigating the phosphorylation at two distinct sites (S171 and S216) that alter chromatin binding and ERα-regulated gene expression, this study uncovered site- and sex-specific effects on hormone regulation, body composition, reproductive function, uterine responses, tissue-specific ERα expression, and systemic metabolism. Unlike prior studies that focused on a single phosphorylation site mutation or larger ERα deletion mutations, this study directly compared two critical ERα phosphorylation sites *in vivo*, analyzing multiple physiological systems. In addition, whereas the previously reported Esr1 S216A mutation was evaluated in mice with a mixed C57B6:129 genetic background,^32^ the present study utilized S216A mice that underwent speed congenic backcrossing and now exist on a pure C57BL/6J background. Preliminary observations found that the pure background C57B6 mice exhibited distinct phenotypes compared with the mixed background C57B6:129 mice. The present study reveals how specific phosphorylation events modulate ERα function, providing new insights into estrogen signaling and potential therapeutic targets for metabolic, reproductive, skeletal, and muscular disorders.

One of the most profound consequences of global and tissue-specific ERα deletion is on the reproductive system, demonstrated by infertility of male and female ERKO mice and infertility in ERα tissue-specific deletion in neurons, adipocytes, pituitary gonadotrophs, and uterine epithelial cells.^46^^,^^47^^,^^48^^,^^49^ Similarly, ERα domain-specific deletions of the DNA-binding or ligand-binding domains resulted in infertility in heterozygous females but not heterozygous males.^23^^,^^24^ The specific contribution of ERα phosphorylation to fertility remains underexplored. Point mutation at phosphorylation site S122 to alanine in mice did not impact fertility in either sex.^30^^,^^31^ In contrast, the present study revealed that S216 phosphorylation, but not S171 phosphorylation, was critical for maintaining female fertility, as S216A females exhibited markedly reduced litter sizes. Over the six-month breeding period, S216A females exhibited consistently reduced litter sizes compared with WT females with no evidence of a progressive decline in fertility over time. Importantly, the length and pattern of the estrous cycle were not altered by the S171A or S216A mutations, indicating that the subfertility observed in S216A females was not due to disruptions in cycle regularity. These findings highlight the sex- and site-specific effects of ERα phosphorylation on fertility regulation, as loss of S171 phosphorylation did not affect fertility in either sex, nor did the loss of S216 phosphorylation in males. The reduced E2 response in the uteri of ovariectomized S216A females likely contributed to the subfertility phenotype in S216A females. S216A females exhibited reduced E2-induced uterine weight increases and suppressed E2-induced gene expression in the myometrium. Impaired E2 signaling in the myometrium may impair essential events such as the adequate expression of oxytocin receptors in myocytes, potentially causing weakened contractions, delayed or obstructed labor (dystocia), and incomplete delivery.^55^^,^^56^ This may explain the several incidences of dystocia-related maternal death in S216A females (data not shown).

The NanoString CosMx spatial transcriptomics platform, which enables single-cell resolution of a 1,000-gene panel, revealed suppressed E2-induced transcriptional programs in S216A uterine SMCs, including pathways for contraction, ECM organization, and angiogenesis. These changes suggest impaired myometrial remodeling and contractility, potentially contributing to dystocia (data not shown) and the observed subfertility in S216A females. The limitation of this spatial transcriptomics study was that spatial transcriptomics was performed with a single sample per group. Replication in larger cohorts will be necessary to validate and extend these findings.

ERα plays a critical role in maintaining bone homeostasis by suppressing osteoclast-mediated bone resorption and promoting osteoblast-mediated bone formation, thereby regulating bone density and skeletal integrity.^57^^,^^58^^,^^59^^,^^60^ Although ERKO mice exhibit elevated circulating sex steroids due to disrupted HPG feedback, their skeletal phenotypes remain highly informative. Female ERKO mice display reduced cortical bone mass and diminished responsiveness to estrogen replacement, indicating a critical role for ERα in maintaining skeletal homeostasis. In the NERKI model, where ERα is unable to bind DNA, cortical bone mass is similarly reduced while trabecular bone is preserved, implicating classical ERα signaling specifically in cortical bone regulation. Moreover, the AF-1 domain of ERα is required for estrogen’s anabolic effects on trabecular bone, but appears dispensable for cortical bone, suggesting domain-specific mechanisms through which ERα differentially regulates skeletal compartments. Targeted deletion of ERα in osteoblasts, osteoblast progenitors, or osteoclasts resulted in significant bone loss, emphasizing the cell-type-specific roles of ERα in skeletal regulation.^61^^,^^62^^,^^63^ The present study expands understanding of the role of ERα in bone by examining the impact of ERα phosphorylation-site-specific mutations. S171A females exhibited increased femoral cortical BMD, whereas S216A females displayed reduced cortical BMD; no significant changes in BMD were observed in S216A or S171A males. Prior studies using the S122A mouse model revealed no BMD changes under basal conditions^30^^,^^31^; however, following orchidectomy and administration of E2, characteristic E2-induced cortical thickening was significantly diminished in S122A males compared with wild-type males. Taken together, findings from the present study and previous studies identify a sex-dependent role for site-specific ERα phosphorylation in regulating bone.

ERα limits fat accumulation by suppressing lipogenesis in white adipose tissue and liver, promoting fatty acid oxidation, and enhancing insulin sensitivity through upregulation of GLUT4 in skeletal muscle. These actions reduce adiposity and preserve lean mass in both sexes, as shown in ERKO models.^64^ The present study examined the site-specific effects of ERα phosphorylation on body composition. Only male S171A and S216A mice exhibited reduced adiposity. Lean mass was increased in S171A females and S216A males, whereas S122A KI mice showed no significant changes in adipose or lean mass in females and a decline in lean mass in males at 9 months.^30^^,^^31^These findings contrast with those of ERKO mice, which showed increased adiposity in both sexes, and highlight the site- and sex-specific roles of S171 and S216 in regulating fat and lean mass.

This study found that ERα phosphorylation at S171 and S216 influences receptor protein levels in a manner that depends on sex, tissue type, and phosphorylation site. These effects may result from changes in ERα conformation that alter interactions with co-regulators and affect protein stability.^7^^,^^65^^,^^66^^,^^67^ These results suggested that regulation of ERα protein levels is controlled by complex, context-dependent mechanisms that will be explored in future studies. The observed variations in ERα protein levels across different tissues and sexes highlight the importance of investigating how site-specific phosphorylation impacts receptor abundance in various physiological contexts.

The findings from this study demonstrate that site-specific phosphorylation of ERα directs tissue-specific regulation of E2 responses. Phosphorylation of ERα modulates estrogen signaling distinctly from the complete loss-of-function phenotypes observed in ERKO. In the S216A KI male and female mice, the ∼2-fold increase in circulating E2 levels and the impaired uterotrophic response in females suggest partial estrogen resistance. In contrast, S171A mice exhibited no change in systemic E2, but did display sex-dependent alterations in body composition and plasma metabolites, indicating a role for S171 phosphorylation in the modulation of E2 effect on metabolism and growth. This phosphorylation site-dependent differential regulation indicates a critical role for ERα phosphorylation in modulating estrogen resistance and estrogenicity.

The more pronounced phenotypes observed in S216A females relative to males indicate a sex-dependent role for ERα phosphorylation at Ser216. However, the molecular basis for this sex specificity is unknown. As no commercially available phospho-specific antibody exists for Esr1 Ser216, a direct assessment of S216 phosphorylation levels in male versus female tissues was not possible. In male mice, it is possible that sex-specific compensatory mechanisms may mitigate the impact of impaired ERα signaling detected in female mice. For instance, androgens acting through the androgen receptor (AR) may functionally compensate for impaired ERα signaling in males. In bone, AR deletion in osteoblast-lineage cells leads to reduced trabecular bone, whereas ERα deletion does not alter cancellous bone in males, suggesting that AR may compensate against impaired ERα input in specific contexts.^68^ Elucidating these potential compensatory pathways will be critical for defining the mechanisms underlying the observed sex-specific effects in the S216A animals.

The findings presented in this report demonstrate the profound influence of single-site ERα phosphorylation on hormone regulation, body composition, fertility, uterine responses, tissue-specific ERα protein expression, and metabolic homeostasis. The results highlight how subtle post-translational modifications may direct complex, sex-specific physiological processes. These findings also illustrate the unique and critical roles of site-specific ERα phosphorylation in modulating ERα activity, providing a new understanding beyond ERα deletion or domain-level disruptions.

### Limitations of the study

While this study provides convincing evidence that phosphorylation at S171 and S216 modulates ERα function, several limitations in the study need further investigation. A limitation of this study is that WT, S171A, and S216A mice were maintained as separate colonies and direct comparisons were made between these colonies rather than comparisons of littermates for each genotype. The absence of littermate controls introduces potential background or colony-specific effects that may impact direct genotype comparisons. To reduce such bias, all mice were age-matched and housed under identical environmental and handling conditions with an identical diet. All female mice used for ERα protein expression analysis in this study were staged at diestrus. Because circulating hormone concentrations vary across the estrous cycle, the ERα protein measurement at only diestrus may not reflect ERα protein levels that might occur at different stages of the cycle. Future studies will quantify systemic hormone levels and ERα protein/phosphorylation status (including pS171 and pS216) at estrus, metestrus, diestrus, and proestrus to determine how dynamic hormonal fluctuations modulate ERα signaling. Furthermore, a limitation of this study is that sex steroids were quantified using immunoassays rather than mass-spectrometry-based methods. The immunoassay platform may lack sensitivity in the low range of E2, particularly in males, and reported values (∼50 pg/mL) may overestimate true biological concentrations. Future studies should incorporate liquid chromatography-tandem mass spectrometry assays to validate key findings in this physiological range. Although this study identified tissue- and sex-specific alterations in ERα protein levels associated with distinct phosphorylation sites, the underlying mechanisms remain undefined. Further investigation is required to elucidate how site-specific phosphorylation influences ERα transcription and ERα protein stability in a tissue-dependent manner. Computational predictions suggested that conformational changes in ERα are induced by phosphorylation; however, direct experimental evidence of these predicted shifts remains to be determined. Future structural studies using cryo-electron microscopy could confirm these computational model predictions. Tissue-specific KI models and inducible KI models would help further define the role of ERα phosphorylation in mice by separating direct, localized effects in a time-dependent manner from the systemic influences present in the global KI models used in the present study. Phospho-mimetic point mutations that reproduce the negative charge of a phosphate group (e.g., a serine-to-glutamate mutation) may offer valuable insights into how mimicking constitutive phosphorylation at specific sites impacts ERα function. pSer216 levels and upstream kinase activity were not directly assessed in male versus female tissues in this study. Addressing this limitation will require a comprehensive kinase-profiling strategy, use of a custom high-affinity pSer216-specific antibody that is currently in development, and evaluation in multiple mouse tissues. Tracking aging cohorts or exposing these mice to metabolic or environmental challenges may uncover how altered ERα phosphorylation contributes to aging and estrogen-related diseases. Assessing risks for metabolic disorders (e.g., alcoholic liver disease), osteoporosis, or hormone-dependent cancers would clarify the long-term physiological consequences of phosphorylation-site mutations. The observed downregulation of collagen and extracellular matrix organization in the S216A uterus, while potentially impairing the uterine remodeling required for fertility, may confer benefits in other physiological contexts. Excessive collagen deposition is a sign of fibrotic diseases, such as lung fibrosis. Therefore, the observed downregulation of collagen in the 216A mouse uterus could mitigate fibrotic processes in non-reproductive tissues, suggesting a broader application of interfering with ERα phosphorylation. Finally, pharmacological approaches targeting kinases or phosphatases that regulate ERα phosphorylation at specific sites could determine whether restoring or inhibiting these modifications mitigates or exacerbates associated phenotypes.

## Resource availability

### Lead contact

Requests for further information and resources should be directed to the lead contact, Brian G. Rowan (browan@tulane.edu).

### Materials availability

Materials used in this study are available from the lead contact upon reasonable request.

### Data and code availability

• The NanoString CosMx spatial transcriptomics raw data generated in this study have been deposited in the NCBI Gene Expression Omnibus: GSE291308. The metabolomics dataset is publicly available through the Science DataBank: https://doi.org/10.57760/sciencedb.31490. All remaining data supporting the findings of this study are available from the lead contact upon reasonable request.
• All analysis code follows standard workflows recommended in the Giotto and Seurat guidelines. These scripts are available from the lead contact upon reasonable request.
• Any additional information, including materials, resources, or protocols used in this study, is available from the lead contact upon reasonable request.

## Acknowledgments

We would like to thank Dr. Masahiko Negishi from NIEHS for providing us the B6.Cg-Esr1^tm1Neg^/J mice. BioRender was used to generate the graphical abstract, license: Zou, B. (2025). https://BioRender.com/t06b466.

## Author contributions

B.Z. performed the majority of experiments, analyzed data, and drafted the manuscript. J.W. and S.F. contributed to immunohistochemistry. M.B.L. and Z.Y. contributed to micro-CT experiments. W.L. conducted NanoString CosMx spatial transcriptomics data analysis. M.J.J. and C.H.S. conducted plasma hormone measurements. R.B. performed pathological assessments of tissues. P.K., C.Z., and A.L. conducted plasma metabolite measurements. M.A. and B.G.R. coordinated the study and supervised the experimental design, analyzed data, and wrote the manuscript.

## Declaration of interests

The authors declare no conflicts of interest.

## STAR★Methods

### Key resources table

**Table:** 

REAGENT or RESOURCE	SOURCE	IDENTIFIER
**Antibodies**		

Progesterone Receptor A/B (C89F7)	Cell Signaling Technology	#3153; RRID: AB_1031219
Ki-67	Biocare Medical	CRM325A; RRID: AB_2721189
Anti-Estrogen Receptor α	Millipore Sigma	06–935; RRID: AB_310305
Phospho-Estrogen Receptor α (Ser167)	Cell Signaling Technology	#64508; RRID: AB_2799660
Negative Control Rabbit IgG	Biocare Medical	NC495 AA

**Chemicals, peptides, and recombinant proteins**		

Xylenes	Fisher Chemical	X3P-1GAL
Hematoxylin	Biocare Medical	CATHE-M
Eosin	Richard-Allan Scientific	7111
Rodent Decloaker	Biocare Medical	RD913
Sesame oil	Millipore Sigma	S3547-250 ML
17β-estradiol	Millipore Sigma	3301-1 GM

**Critical commercial assays**		

Background Sniper	Biocare Medical	BS966L
MACH 4 HRP Polymer	Biocare Medical	M4U534
ImmPACT DAB	Vector Laboratories	SK-4105
Micromount	Leica	3801731
Testosterone ELISA kit	IBL-America	IB79106
MILLIPLEX MAP Multi-Species Hormone Magnetic Bead Panel	Millipore Sigma	MSHMAG-21K
MILLIPLEX MAP Mouse Pituitary Magnetic Bead Panel	Millipore Sigma	MPTMAG-49K
MiniCollect K2EDTA tubes	Greiner Bio-One	450480

**Deposited data**		

NanoString CosMx spatial transcriptomics raw data	This paper	GSE291308
Metabolomics dataset	This paper	https://doi.org/10.57760/sciencedb.31490

**Experimental models: Organisms/strains**		

C57BL/6N Esr1 S171A	Biocytogen	N/A
B6.Cg-Esr1^tm1Neg^/J	The Jackson Laboratory	RRID:IMSR_JAX:036196

**Oligonucleotides**		

Primer: F-CTGCTTGTGGATCAGATATAAGCT; R-GTCTTATCTTGCCAGGGGGAAGCAG	This paper	N/A

**Software and algorithms**		

QuPath (v0.4.4)	Bankhead et al.^69^	https://qupath.github.io/
Analyze 14.0	AnalyzeDirect, Inc.	https://analyzedirect.com/analyze14/
Cellpose	Stringer et al.^70^	https://github.com/mouseland/cellpose
Seurat (5.1.0)	Hao et al.^71^	https://satijalab.org/seurat/
Giotto (4.2.0)	Dries et al.^72^	https://github.com/drieslab/Giotto
STRING-db v12.0	Szklarczyk et al.^73^	https://string-db.org/
Prism 10	GraphPad Software	https://www.graphpad.com/features

### Experimental model and study participant details

#### Species and strain background

All experiments were performed using Mus musculus on a C57BL/6 genetic background. The ESR1-S171A and ESR1-S216A phospho-deficient knock-in mouse lines were generated and maintained on a C57BL/6N and C57BL/6J, respectively.

#### Genotypes

WT (Esr1^+/+^); Esr1S171A: serine-to-alanine substitution at residue 171 of ERα; Esr1S216A: serine-to-alanine substitution at residue 216 of ERα.

#### Sex, age, and developmental stage

Both male and female mice were used. Age ranges: 3 months for physiological and metabolic studies.

#### Institutional permissions

All animal experiments were conducted in accordance with NIH guidelines and approved by the Tulane University Institutional Animal Care and Use Committee (IACUC) under protocol #1917. Earlier phases of this project were conducted under protocol #999.

#### Sex as a biological variable

Phosphorylation-deficient ERα mutations exhibited sex-specific effects on physiological outcomes. In females, the S171A mutation increased BMD and lean mass, whereas the S216A mutation decreased BMD with no effect on lean mass. In males, BMD remained unchanged, but the S216A mutation increased lean mass. Both S171A and S216A mutations reduced adipose tissue in males, with no effect observed in females. Impaired fertility was observed only in S216A females.

#### Mice

The S171A knock-in (KI) mice were generated by Biocytogen (Waltham, MA, USA) using CRISPR/Cas9 genome editing to introduce a site-specific mutation in exon 3 of the target gene, replacing the serine codon (AGC) with alanine (GCC), based on transcript NM_007956. The targeting vector was constructed with ∼1.5 kb homologous arms flanking the mutation site to facilitate homologous recombination. Guide RNAs (sgRNAs) were designed using an online CRISPR design tool (http://www.sanger.ac.uk/htgt/wge/) and validated for activity with the Universal CRISPR Activity Assay (UCATM) (Figure S1). The Cas9 protein, sgRNAs, and donor DNA were microinjected into zygotes from C57BL/6 mouse embryos. The zygotes were then implanted into pseudopregnant females for gestation. Founder mice (F0) were screened for successful integration of the mutation by PCR (Primer: F-CTGCTTGTGGATCAGATATAAGCT; R-GTCTTATCTTGCCAGGGGGAAGCAG) and Sanger sequencing. Southern blot analysis was used to confirm the absence of random insertions and ensure specificity at the target site. Confirmed founder mice were bred with wild-type C57BL/6N mice to produce F1 heterozygous offspring. Genotyping of the F1 mice was conducted using junction PCR and Sanger sequencing, complemented by Southern blot analysis with probes targeting the 5′ and 3′ homologous arms. Homozygous S171A mice were obtained by mating heterozygous males and females and confirmed by Sanger sequencing. The Esr1 S216A KI mice^32^ were generated by the laboratory of Dr. Masahiko Negishi (National Institute of Environmental Health Sciences) using a targeting vector to introduce a serine-to-alanine substitution at serine 216 in exon 3. The vector included an ACN self-excising loxP-flanked neomycin resistance cassette and a diphtheria toxin (DTA) gene. Chimeric males were crossed with B6(Cg)-Tyrc-2J/J females to achieve germline transmission and self-excision of the cassette. The resultant B6:129 mixed-background mice were backcrossed onto a C57BL/6J background via speed congenic breeding by Jackson Laboratory, eliminating the Tyrc-2J allele to produce pure C57BL/6J Esr1 S216A mice. All the breeding of wild-type (WT), S171A, and S216A mice was conducted at the vivarium of the Department of Comparative Medicine at Tulane University. Animal maintenance and experimental procedures were carried out following protocols approved by the Tulane Institutional Animal Care and Use Committee (IACUC).

### Method details

#### Alphafold prediction for ERα structure

The sequence of mouse ESR1 (ESR1_MOUSE; UniProt P19785) was retrieved from UniProt, and the canonical estrogen response element (ERE) sequence, described by Véronique et al.,^74^ was incorporated along with zinc ions to replicate the native coordination environment of the ERα zinc-finger domains. Structural predictions of ERα dimers bound to the ERE sequence were performed using AlphaFold 3 (https://alphafoldserver.com/). Model confidence was assessed using predicted local distance difference test (pLDDT) scores generated by the AlphaFold pipeline, and the highest-confidence models were selected for downstream analyses. The resulting AlphaFold models (*cif files)* were visualized using Mol 3D Viewer^19^^,^^75^ to evaluate hydrogen bonding, residue-residue interactions, and phosphorylation-induced conformational changes.

#### Histology

Tissues (bone, mammary gland, heart, testis, liver, kidney, brain, lung, and uterus) were collected from mice and processed for histological analysis and immunohistochemistry as previously described.^76^^,^^77^ Paraffin-embedded tissue sections (4 μm) were mounted on glass slides. For hematoxylin and eosin (H&E) staining, sections were deparaffinized using Xylene (Fisher Chemical, X3P-1GAL), rehydrated, and stained with hematoxylin (Biocare Medical, CATHE-M) and eosin (Richard-Allan Scientific, 7111). Stained sections were subsequently dehydrated, cleared by Xylene (Fisher Chemical, X3P-1GAL), and mounted with Micromount (Leica, 3801731). For immunohistochemistry, deparaffinized by Xylene (Fisher Chemical, X3P-1GAL) and rehydrated sections underwent antigen retrieval using Rodent Decloaker (Biocare Medical, RD913) and nonspecific binding was minimized using Background Sniper (Biocare Medical). Slides were incubated overnight at 4°C with primary antibodies, including anti-PR (1:100, Cell Signaling, C89F7), Ki-67 (1:50, Biocare Medical, CRM325A), anti-ERα (1:1000, Sigma-Aldrich, 06–935) or anti-Rabbit IgG (Biocare Medical, NC495 AA). Following incubation, sections were treated with MACH 4 HRP Polymer (Biocare Medical, M4U534) for 30 min at room temperature. Signal development was achieved using ImmPACT DAB (Vector Laboratories, SK-4105) and visualized under a microscope. Sections were counterstained with hematoxylin, dehydrated, and mounted with Micromount (Leica, 3801731). IHC images were acquired at 200× magnification using EVOS FL Auto Imaging System (Thermo Fisher Scientific), were imported into QuPath (v0.4.4) for analysis.^69^ Five regions of interest (ROIs) were randomly selected from each slide for quantitative assessment. Positive cells within the ROIs were identified using the “Positive Cell Detection” tool, which quantified the percentage of positive nuclei and assigned a staining intensity score to each detected positive cell, ranging from 0 (negative) to 3 (strongly positive). The H-score for each ROI was automatically calculated using the formula: H-score = 3 x percentage of strongly staining nuclei +2 x percentage of moderately staining nuclei + percentage of weakly staining nuclei. For P-S171 staining (Figure 1D), uterine sections from six WT and six S171A females were incubated overnight with a rabbit polyclonal anti-phospho-ERα (Ser167) antibody (Cell Signaling #64508, 1: 800). Whole-slide images acquired at 200 × were imported into QuPath and analyzed with the Positive Pixel Detection function. Five randomly selected ROIs per animal were quantified. The percentage of P-S171-positive area was calculated as: (DAB-positive pixels/total tissue pixels) × 100. Mean values from the five ROIs were used to represent a single biological replicate for statistical comparisons.

#### Plasma hormone measurement

Blood was collected using MiniCollect K2EDTA tubes (Greiner Bio-One, 450480) and centrifuged at 1300 × g for 10 min at 4°C. The plasma was collected, snap-frozen in liquid nitrogen, and stored at −80°C for future analysis. Testosterone levels were quantified using a Testosterone ELISA kit (IBL-America, SKU: IB79106) following the manufacturer’s instructions. Estradiol and progesterone levels were measured using the MILLIPLEX MAP Multi-Species Hormone Magnetic Bead Panel (Millipore Sigma, MSHMAG-21K). Follicle-stimulating hormone (FSH) and luteinizing hormone (LH) levels were assessed with the MILLIPLEX MAP Mouse Pituitary Magnetic Bead Panel - Endocrine Multiplex Assay (Millipore, MPTMAG-49K) according to the manufacturer’s instructions.

#### Body composition analysis

Body composition analysis was performed on 5–7 mice per group using a Quantum GX2 Micro-CT scanner (PerkinElmer). Details of the scanning and quantification protocols are standardized by our lab.^54^ Briefly, mice were anesthetized with isoflurane and scanned using two parameter sets: one for bone mineral density (BMD) quantification (90 kV, 88 μA, 36 acquisitions, 36 reconstructions, 18-s standard scan, Cu 0.06 + Al 0.5 filter) and another for body composition analysis (45 kV, 133 μA, 36 acquisitions, 36 reconstructions, 18-s standard scan, no filter). A hydroxyapatite (HA) phantom (Micro-CT HA D25, QRM, Cat. #70129) containing five densities (0, 50, 200, 800, and 1200 mg HA/cc) was scanned to generate a standard curve for BMD calculations. The resulting output files (.vox) from the scans were imported into Analyze 14.0 software (https://analyzedirect.com/analyze14/) for the segmentation of bone, adipose tissue, and lean tissue based on Hounsfield unit thresholds. Femoral cortical bone thickness was measured at the mid-diaphysis, and cortical BMD was calculated using the phantom-based standard curve. Adipose and lean tissue volumes were converted to mass using densities of 0.95 g/cm^3^ and 1.05 g/cm^3^, respectively, and normalized to body weight to calculate their percentages of total body mass.^78^

#### Fertility assessment

Three-month-old WT male and female mice were initially paired to confirm proven breeder WT males and females by producing a first litter. Following the establishment of the WT proven breeders, these WT mice were subsequently paired with either S171A or S216A mutant mice as described in Figure 3A. Each mating pair was monitored over a six-month observation period to record the fertility parameters, including the number of litters produced, the interval between litters, litter size (pups per litter), and pup sex ratios (female-to-male ratio).

#### Uterotrophic assay

For the uterotrophic assay, three-month-old WT, S171A, or S216A female mice (5–6 animals per group) were ovariectomized (OVX). The mice were allowed to recover for 10 days to stabilize baseline estradiol levels. Following recovery, each mouse received a daily subcutaneous injection of 10 μg/kg 17β-estradiol (E2) (Millipore, 3301-1 GM) dissolved in sesame oil (Sigma, S3547-250 ML) for three days.^26^ Mice were euthanized 24 h after the final injection, and wet uteri were collected and weighed to assess uterine responsiveness. Uteri were then fixed in formalin for subsequent histological analysis.

#### Spatial transcriptomics

Formalin-fixed, paraffin-embedded tissue blocks (*n* = 1) from the uterotrophic assay were sectioned into 4 μm slides that fit in the 15 mm × 20 mm imageable area and sent to NanoString Technologies (Bruker Spatial Biology, Seattle, WA, USA) for spatial transcriptomic analysis using the CosMx Spatial Molecular Imager (CosMx SMI) platform.^79^ This platform utilizes cyclic fluorescent *in situ* hybridization (FISH) chemistry to enable highly multiplexed gene expression profiling. A 1000-gene panel was used for this study, with a detailed list of the included genes provided in the Table S3. The antibodies B2M/CD298, PanCK, CD45, and CD3, along with DAPI, were used as morphological markers. Cell segmentation was performed using NanoString’s machine learning-based algorithm Cellpose,^70^ which detects individual cells across experimental conditions (WT_VC, S171A_VC, S216A_VC, WT_E2, S171A_E2, S216A_E2) (Figure S5D). Raw gene expression matrices and spatial coordinates were generated for downstream analysis. Data normalization was conducted using the SCTransform method with the Nanostring assay. Then, principal component analysis (PCA) and the top 30 principal components (PCs) were used to conduct nearest-neighbor graphs via the Seurat’s (5.1.0) FindNeighbors function, followed by clustering analysis using the FindClusters function.^71^ Uniform Manifold Approximation and Projection (UMAP) was subsequently applied using the top 30 PCs for visualization of clusters in low-dimensional space. The clusters were annotated using the previously established markers. The normalized total transcript count was visualized using Giotto’s (4.2.0) spatPlot2D tool.^72^ Differential expression analysis was performed using Seurat’s FindMarkers tool to identify transcriptional changes between genotypes (WT, S171A, S216A) and treatments (vehicle vs. E2). Comparisons included both global analyses across all cells and cell type-specific assessments. Adjusted *p*-values (Benjamini-Hochberg method) were calculated to correct for multiple testing, and results were filtered for genes with ≥25% log2 fold-change and detection in ≥10% of cells. A curated panel of 18 genes, from the 1000-gene panel, encoding key regulators of smooth muscle contraction (Acta2, Actg2, Myh11, Myl4, Myl7, Myl9, Myl12a, Myl12b, Tpm1, Tpm2, Tnnc1, Tcap, Tagln, Calm1, Calm2, Calm3, Cacna1c, Ryr2) was analyzed. Single-cell contraction scores were computed using Seurat’s AddModuleScore (v5.1.0). 5 control genes were randomly selected by the function from expression-matched bins used for normalization. Scores were calculated on SCTransform-normalized data and visualized through Seurat’s spatial feature mapping tool (ImageFeaturePlot) to assess myometrial contractile potential across genotypes. Pathway enrichment analysis was conducted using STRING-db v12.0^73^ (https://string-db.org/), with Gene Ontology (GO), KEGG, WikiPathways, and Reactome gene sets. Pathways were considered significant at a false discovery rate (FDR) < 0.05.

#### Metabolomics

Plasma was collected following the same procedure used for plasma hormone measurements. The plasma samples were stored on dry ice and shipped to Gigantest, Inc. (Baltimore, MD, USA) for metabolomic analysis. Metabolites were extracted from plasma samples across all experimental groups using 100% liquid chromatography-mass spectrometry (LC-MS) grade methanol, adhering to an 80:20 methanol-to-sample (vol/vol) ratio as described in previous studies.^80^^,^^81^^,^^82^ Following extraction, methanol and water were evaporated from the samples, leaving behind dried metabolites, which were subsequently reconstituted in a solution of 50% acetonitrile (vol/vol) in MS-grade water for metabolomic analysis. For data acquisition, a Thermo Scientific IQX Mass Spectrometer was used in conjunction with a Vanquish UPHLC system. The samples were maintained at 4°C throughout the LC process, and an injection volume of 2 μL was utilized for each sample. A 15-min reverse-phase chromatography protocol was applied using a Discovery HSF5 reverse-phase column (Sigma). The mobile phase consisted of 0.1% formic acid in MS-grade water for the aqueous phase and 0.1% formic acid in acetonitrile for the organic phase. Prior to data acquisition, instrument calibration was performed to ensure optimal sensitivity and accuracy. The final metabolite intensity values were obtained by integrating chromatographic peak areas, with normalization performed relative to the sample protein concentration.

### Quantification and statistical analysis

Statistical analyses were performed using GraphPad Prism 10. The *n* values and statistical details are provided in figure legends. Comparisons between the two groups were conducted using unpaired two-tailed Student’s t-tests. For comparisons involving more than two groups, one-way or two-way analysis of variance (ANOVA) was performed as appropriate. When ANOVA indicated a significant effect, Tukey’s honestly significant difference (HSD) test was applied post hoc to correct for multiple comparisons. Statistical significance was defined as *p* < 0.05.

In Figures 1E–1J, 2B–2D, 2F, 2H, 2J, 2L, 2N, 7C–7F, S2B–S2D, S2F, S2H, S2J, S2L, S2N, S2P, and S13, n represents individual mice, as specified in the corresponding figure legends. Statistical analyses were conducted using GraphPad Prism 10 with one-way ANOVA, and detailed statistical parameters are provided in the respective legends. In Figures 3 and S3, n represents individual mating pairs, as indicated. One-way ANOVA was used for analysis in GraphPad Prism 10, with complete statistical details included in the figure legends. In Figures 4C, 4D, 4F, S4A, and S4C, n represents individual mice. Analyses were performed using two-way ANOVA in GraphPad Prism 10. Figure legends include full statistical details, including factors, post hoc tests, and significance thresholds. For all datasets, definitions of center (mean), dispersion and precision measures (SEM) are reported in the respective result and figure legends.

## Supplemental information

Document S1. Figures S1–S13

Table S1. Complete two-way ANOVA and Tukey post hoc results of Figure 4

Table S2. Plasma metabolites levels

Table S3. 1000-plex gene panel

Table S4. Full names of the abbreviated pathways in Figure 5
