# Site-specific ERα phosphorylation determines sex-dependent metabolic, reproductive, and body-compositional phenotypes in mice

**DOI:** 10.1016/j.isci.2025.114413

**Published:** 2025-12-20

**Authors:** Binghao Zou, Jarvis Williams, Madeleine B. Landau, Weiqiang Lin, Sallie Fell, Ziqi Yang, MaryJane Jones, Robert Blair, Chad H. Steele, Pratik Khare, Cissy Zhang, Anne Le, Muralidharan Anbalagan, Brian G. Rowan

## Main text

(iScience *28*, 114112; December 19, 2025)

Due to a typesetter error, in the originally published version of this article, Muralidharan Anbalagan was not listed as a corresponding author. This has been corrected online so that Dr. Anbalagan’s corresponding author status is now properly indicated. The production team apologizes for this error.

