# Supplementary material for: Site-specific ERα phosphorylation determines sex-dependent metabolic, reproductive, and body-compositional phenotypes in mice
Source: iScience. 2025 Nov 19;28(12):114112. doi: 10.1016/j.isci.2025.114112 (PMC12719750; doi:10.1016/j.isci.2025.114112)
Supplement: Document S1. Figures S1–S13 [file mmc1.pdf]

iScience, Volume *28*

Supplemental information

Site-specific ERα phosphorylation determines sex-dependent metabolic, reproductive, and body-compositional phenotypes in mice

Binghao Zou, Jarvis Williams, Madeleine B. Landau, Weiqiang Lin, Sallie Fell, Ziqi Yang, MaryJane Jones, Robert Blair, Chad H. Steele, Pratik Khare, Cissy Zhang, Anne Le, Muralidharan Anbalagan, and Brian G. Rowan

A

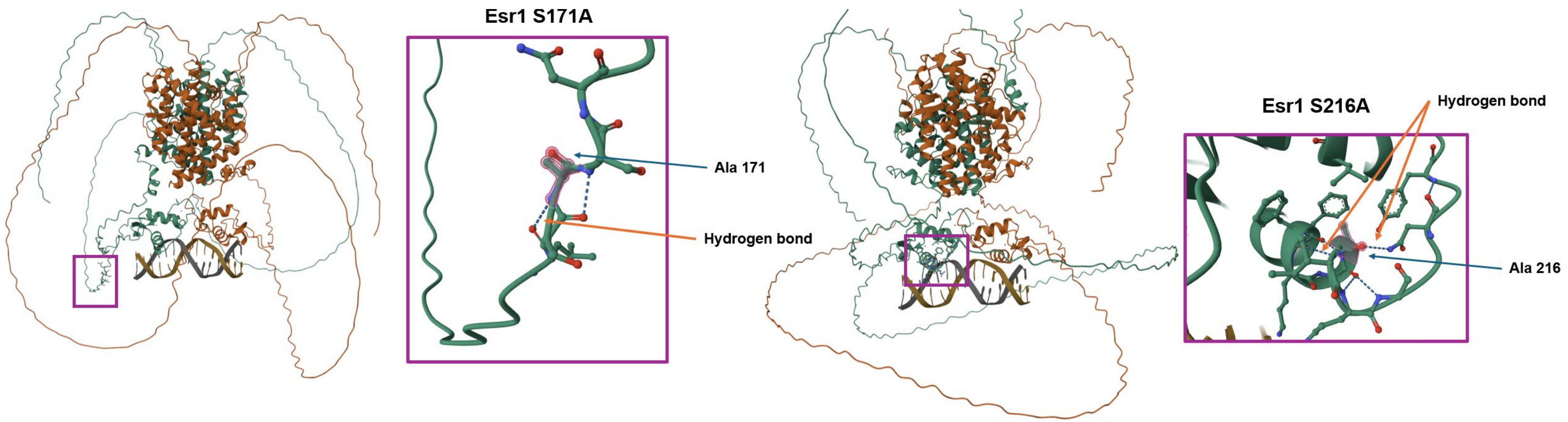

C

5'Guide	Sequence (5'-3')
Guide #1	ATCCTTATGTTATTGTTATC GGG
Guide #2	TTATTGTTATCGGGCAACCC AGG
Guide #3	TCCAACCTTCAAAACCTTCG TGG
Guide #4	ACCTTCAAAACCTTCGTGGA AGG
Guide #5	TTGCGTTCACTTTGTCAAAT GGG
Guide #6	AATCCATGCGAGCGGTCCTC TGG
Guide #7	GAGGACCGCTCGCATGGATT TGG
Guide #8	CAAGCCCAAATCCATGCGAG CGG

3'Guide	Sequence (5'-3')
Guide #9	CCACTAGAAGAATTGGTAAC TGG
Guide #10	AACTGGGTCTCTAGTGTACA AGG
Guide #11	ATCAGAACATAATATTTACC TGG
Guide #12	CTGATGGGGTGTCCCTCATA AGG
Guide #13	TATAAGTACACAGTACTGAT GGG
Guide #14	TCTGTATCGTGTGACAGTAC TGG
Guide #15	TGTGCACTAAAGTCCTTGAC AGG
Guide #16	TCTGATATGGGCTAACAACC AGG

B

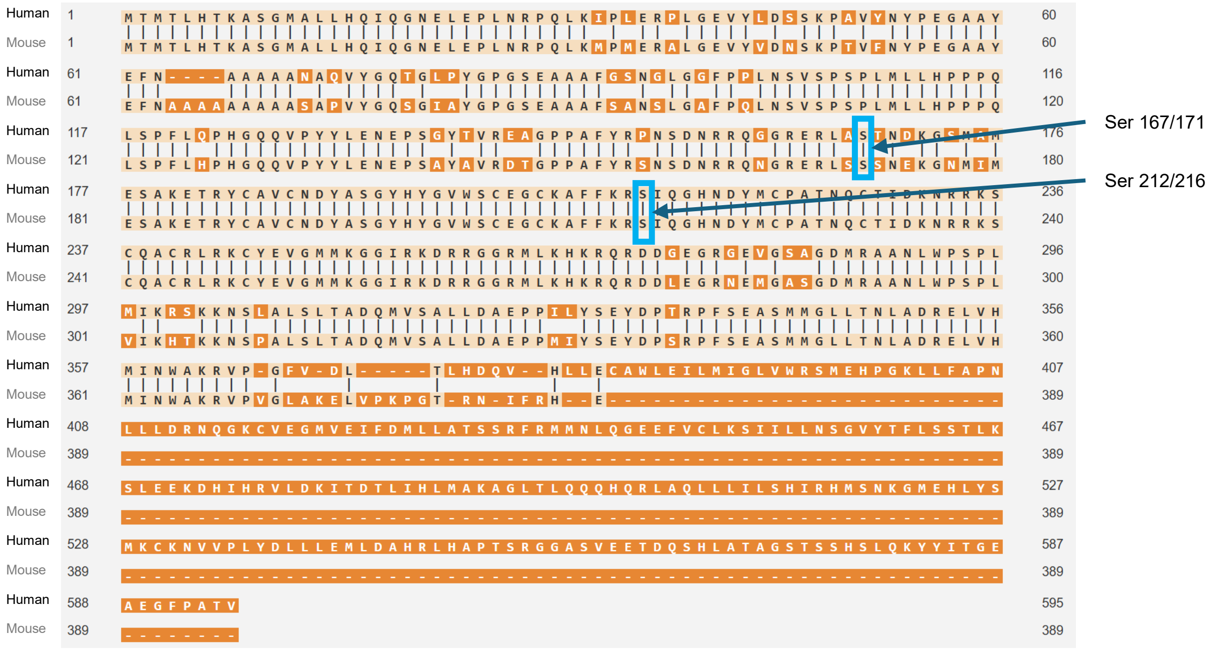

D

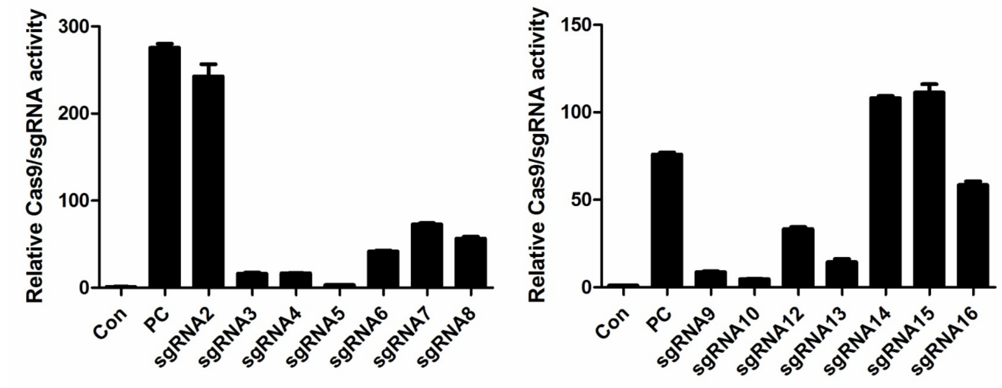

Supplementary Figure 1. Design of CRISPR/Cas9 genomic editing targeting mouse ESR1, related to Figure 1. (A) Predicted 3D structures of Esr1 S171A and Esr1 S216A generated by AlphaFold3, with magnified views of the regions surrounding phosphorylation sites S171 and S216. Key molecular features are highlighted including hydrogen bonds (orange arrows) and alanine residues (blue arrows). This figure was generated using the Mol* 3D Viewer[1]. (B) Sequence alignment of human (top) and mouse (bottom) ERα with boxed regions indicating serine residues corresponding to Ser167/171 and Ser212/216. (C) List of 5′- and 3′-guide RNA (sgRNA) sequences used in the CRISPR/Cas9 editing strategy showing the sequence of each sgRNA. (D) Bar graphs depicting relative Cas9/sgRNA activity for the tested sgRNAs as determined by a universal CRISPR activity assay.

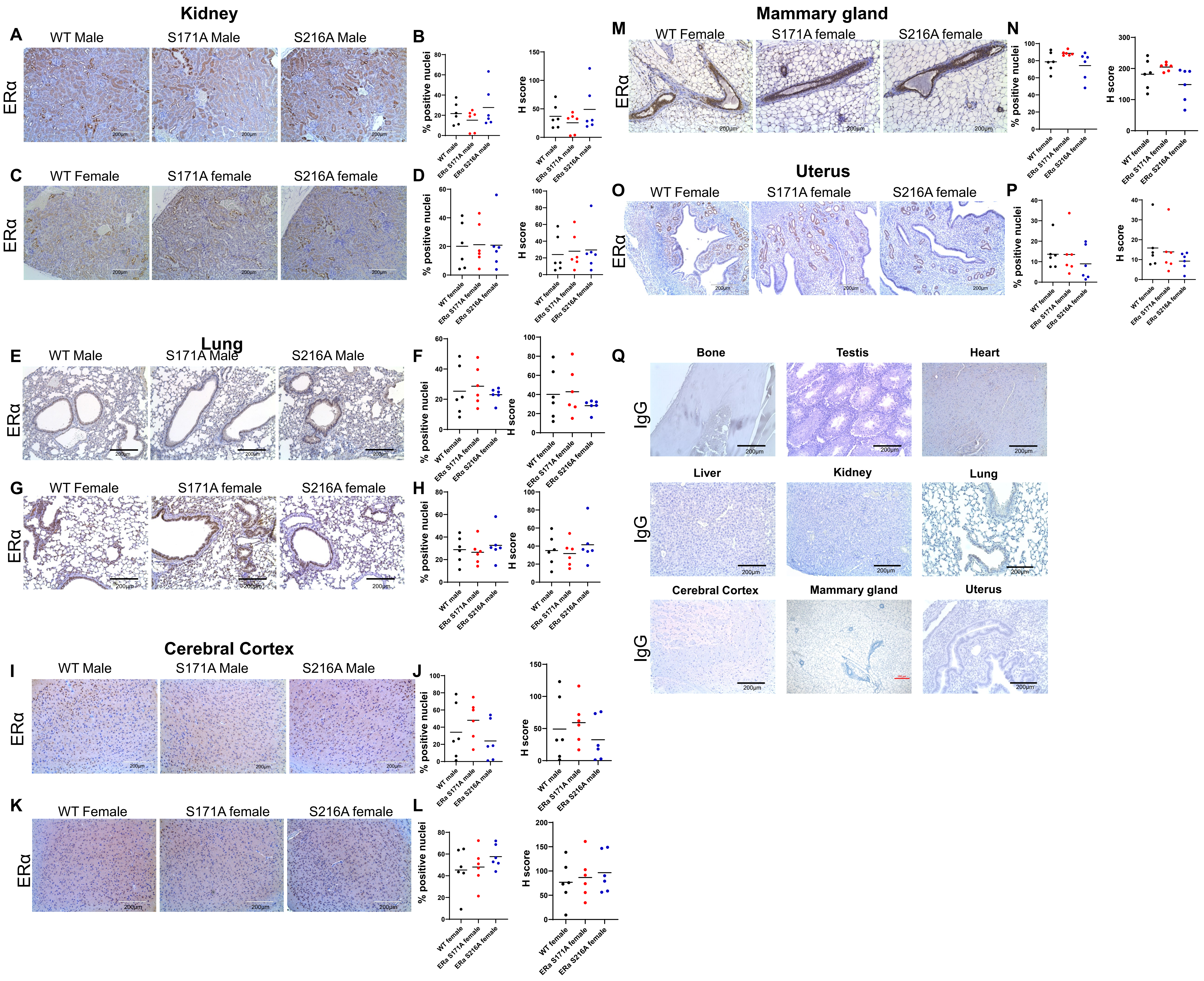
Kidney
A
WT Male
S171A Male
S216A Male
ERα
200μm
B
% positive nuclei
H score
WT male
ERα S171A male
ERα S216A male
C
WT Female
S171A female
S216A female
D
WT female
ERα S171A female
ERα S216A female
Lung
E
F
G
H
Cerebral Cortex
I
J
K
L
Mammary gland
M
N
Uterus
O
P
Q
Bone
Testis
Heart
Liver
Kidney
Lung
Cerebral Cortex
Mammary gland
Uterus
IgG

Supplementary Figure 2. IHC analysis of ERα in the kidney, lung, cerebral cortex, mammary gland, and uterus of WT, S171A, and S216A mice, related to Figure 2. (A-P) Representative IHC images are shown for male and female mice with corresponding quantitation of ERα expression in scatter plots. Panels illustrate ERα staining in the kidney, lung, cerebral cortex, mammary gland and uterus. Scale bars represent 200 µm. Statistical analysis was performed using one-way ANOVA. Data are presented as individual data points with the mean. n = 6 mice per group. (Q) IgG negative control immunostaining in bone, testis, heart, liver, kidney, lung, cerebral cortex, mammary gland, and uterus. Scale bars represent 200 µm.

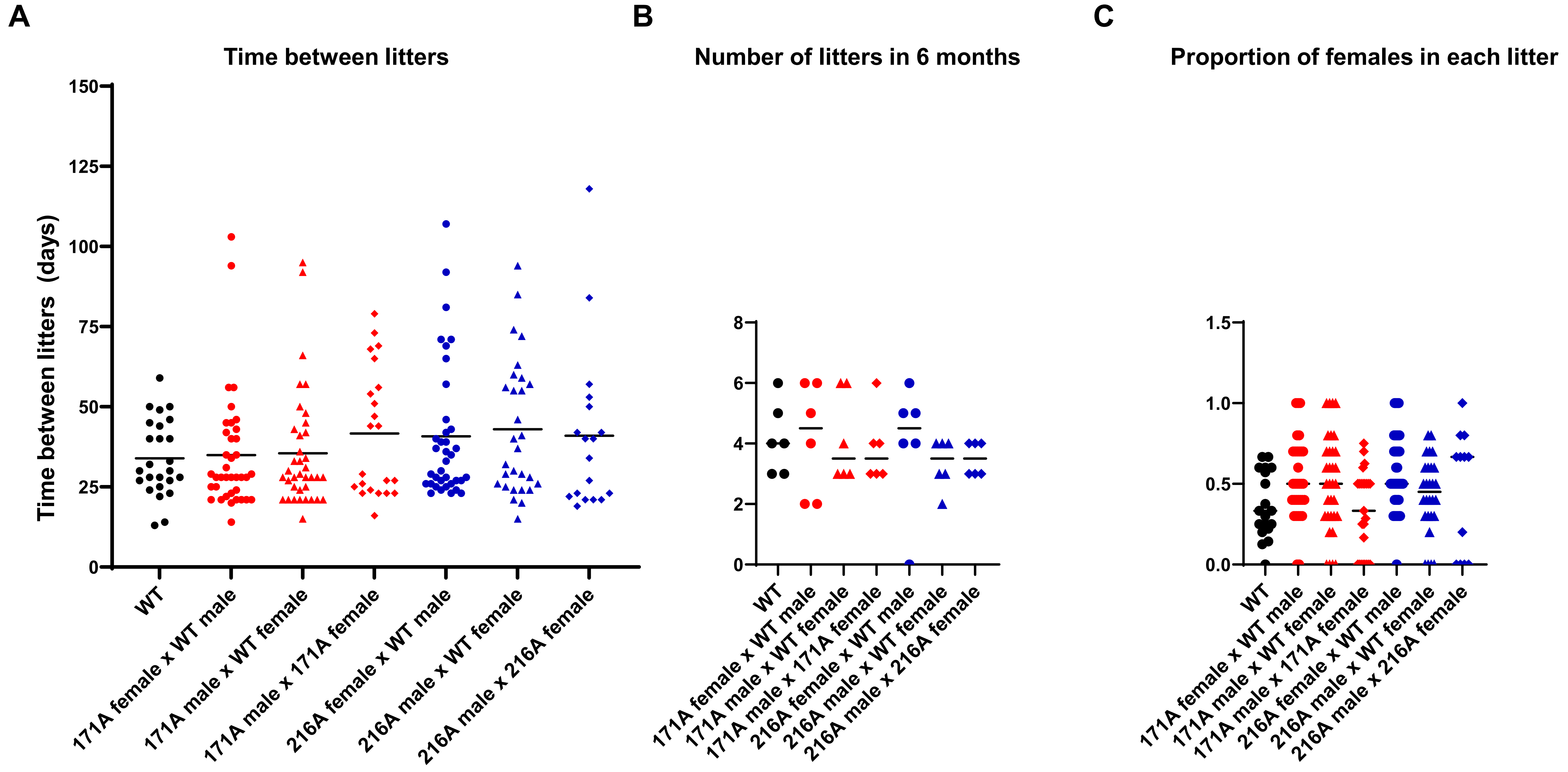
A
Time between litters
Time between litters (days)
150
125
100
75
50
25
0
WT
171A female x WT male
171A male x WT female
171A male x 171A female
216A female x WT male
216A male x WT female
216A male x 216A female
B
Number of litters in 6 months
8
6
4
2
0
C
Proportion of females in each litter
1.5
1.0
0.5
0.0

Supplementary Figure 3. Fertility parameters in WT, S171A, and S216A ERα mice, related to Figure 3. (A) Scatter plots of the interval (in days) between litters for each mating pair. (B) Scatter plots of the total number of litters produced over a 6-month fertility study period. (C) Scatter plots of the proportion of female offspring within each litter. Data are presented as individual data points with the mean. n = 6 mating pairs per group. Statistical analysis was performed using one-way ANOVA.

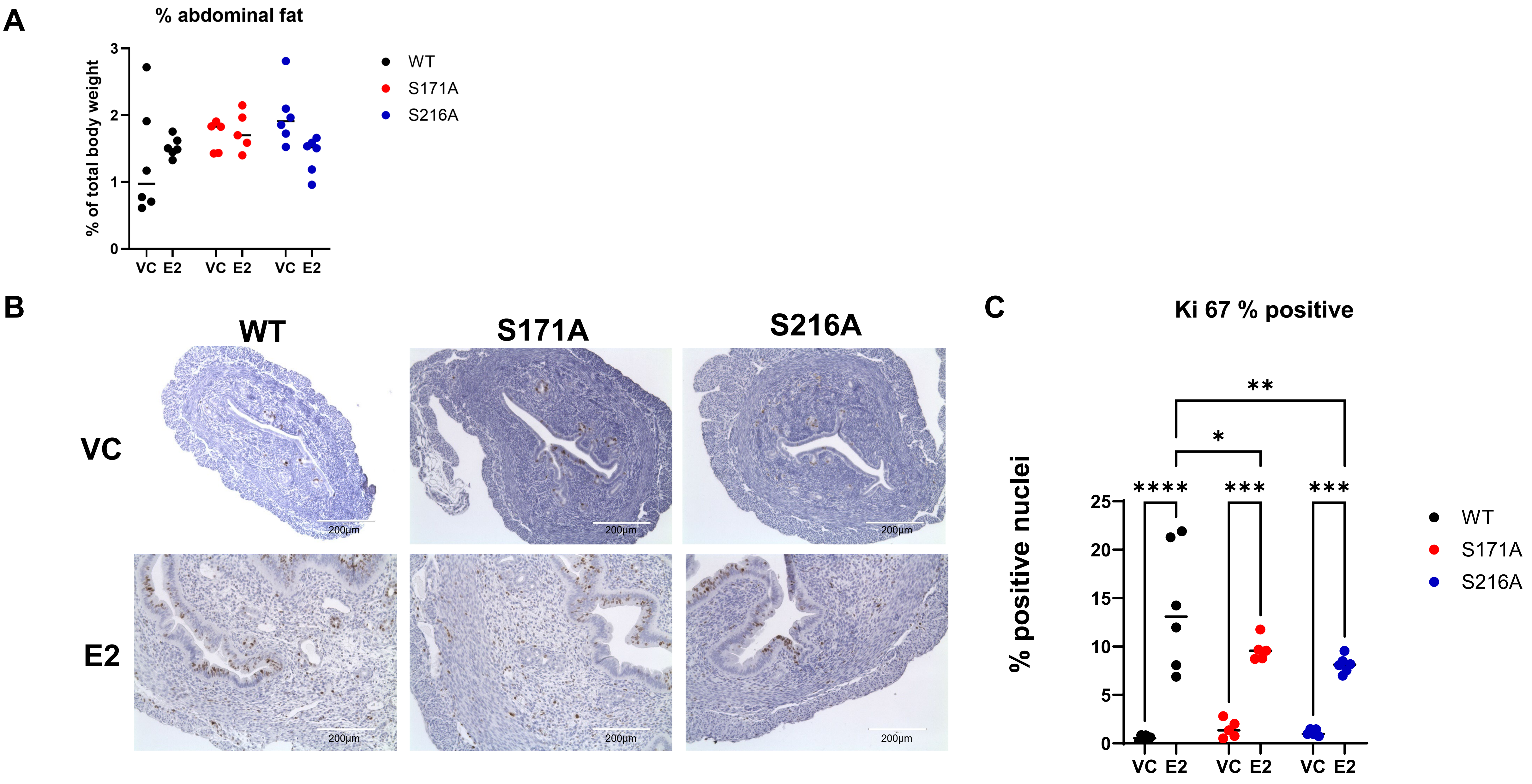
A
% abdominal fat
% of total body weight
0
1
2
3
VC E2 VC E2 VC E2
WT
S171A
S216A
B
WT
S171A
S216A
VC
E2
200µm
C
Ki 67 % positive
% positive nuclei
0
5
10
15
20
25
****
***
***
*
**
VC E2 VC E2 VC E2
WT
S171A
S216A

Supplementary Figure 4. Immunohistochemical detection of Ki67 in uterine cross-sections from the experiment in Figure 4 using ovariectomized mice incubated with vehicle control (VC) or E2 (daily subcutaneous injection of 10 µg/kg E2 dissolved in sesame oil for 72 hours), related to Figure 4. (A) Quantitative analysis of abdominal fat weight. (B) Representative images of Ki67 immunostaining in uterine cross-sections from WT, S171A, and S216A mice following VC or E2 treatment. Scale bars: 200 µm. (C) Scatter plot depicting the percentage of Ki67-positive nuclei in each group. Statistical analysis was performed using two-way ANOVA followed by Tukey's post hoc test. Significance levels are indicated as follows: *$P < 0.05$, **$P < 0.01$, ***$P < 0.001$, ****$P < 0.0001$."

A

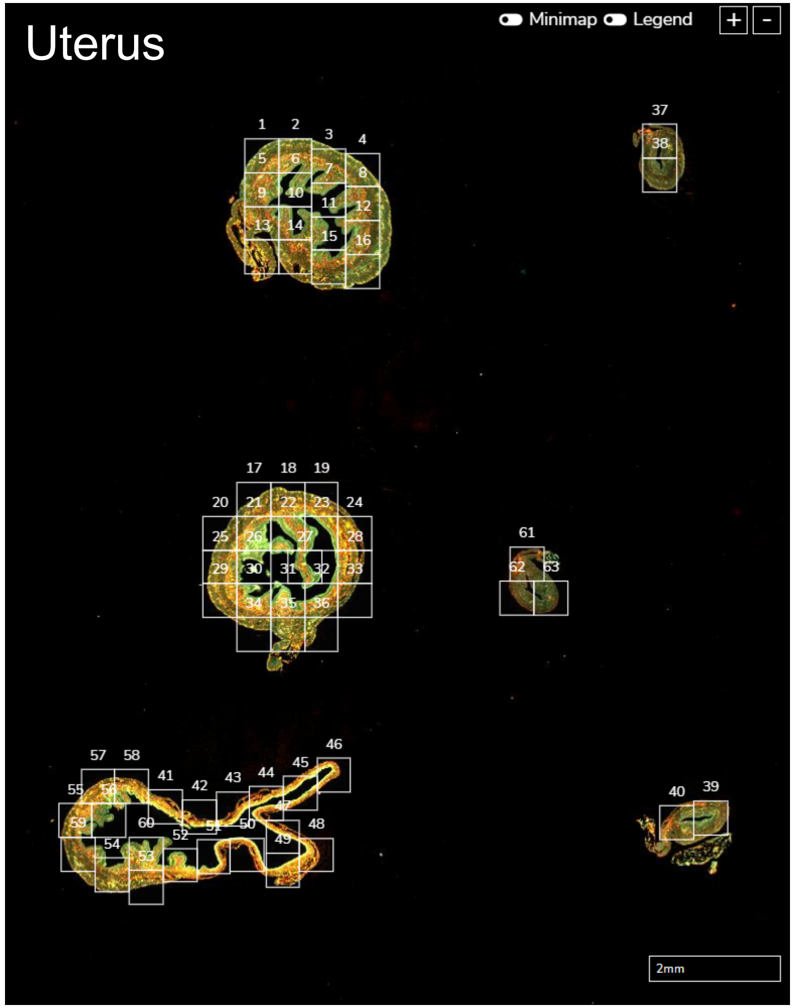

B

Number of FOVs	63
Total Effective Tissue Area (mm^2)	8.39
Mean Single Cell Size (um^2)	75.2
Number of Cells in Final Analysis	111,612
Total Transcripts Detected in Cells	15,208,280
Mean Transcripts per Cell	133
90-Percentile of Transcripts per Cell	302
Maximum Transcripts per Cell	1,742
Mean Unique Genes Per Cell	71
Mean transcripts per um^2	1.8
Mean Negative per Plex per Cell	0.014
Mean False Codes per Plex per Cell	0.004

C

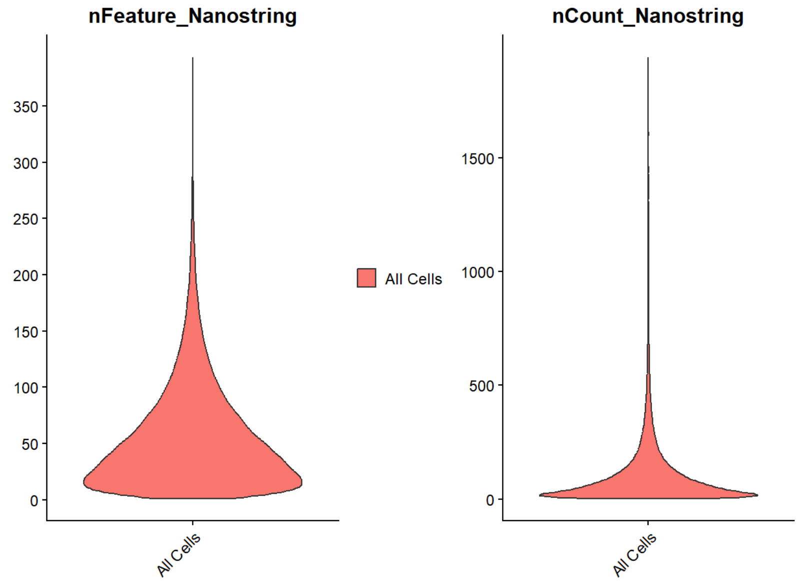

D

Machine learning-based cell segmentation

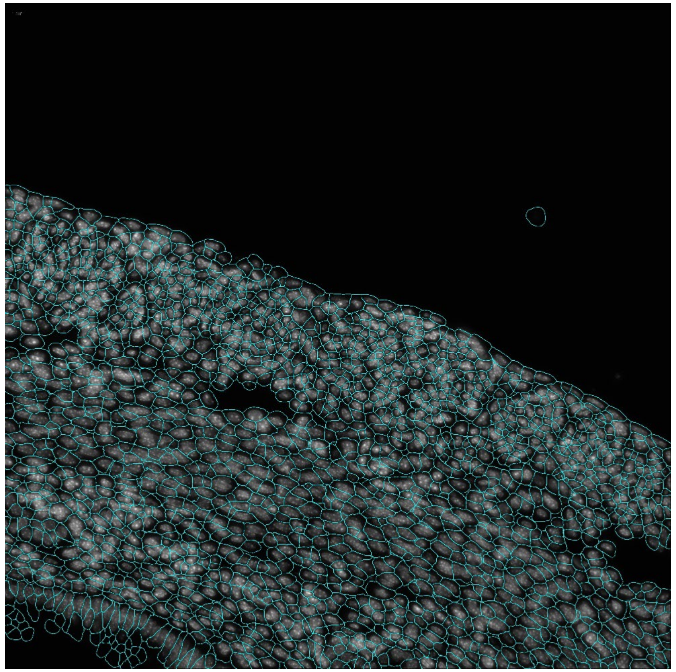

Supplementary Figure 5. Quality control for Nanostring Cosmx spatial transcriptomics, related to Figure 5. (A) Scan of the uterine tissue section (63 fields of view) processed for spatial transcriptomic analysis. Scale bars represent 2 mm. (B) Key quality control metrics (C) Feature abundance (nFeatures) and transcript counts (nCount) across all cells (D) Machine learning-based single-cell segmentation (Cellpose algorithm) of uterine tissue for downstream analysis.

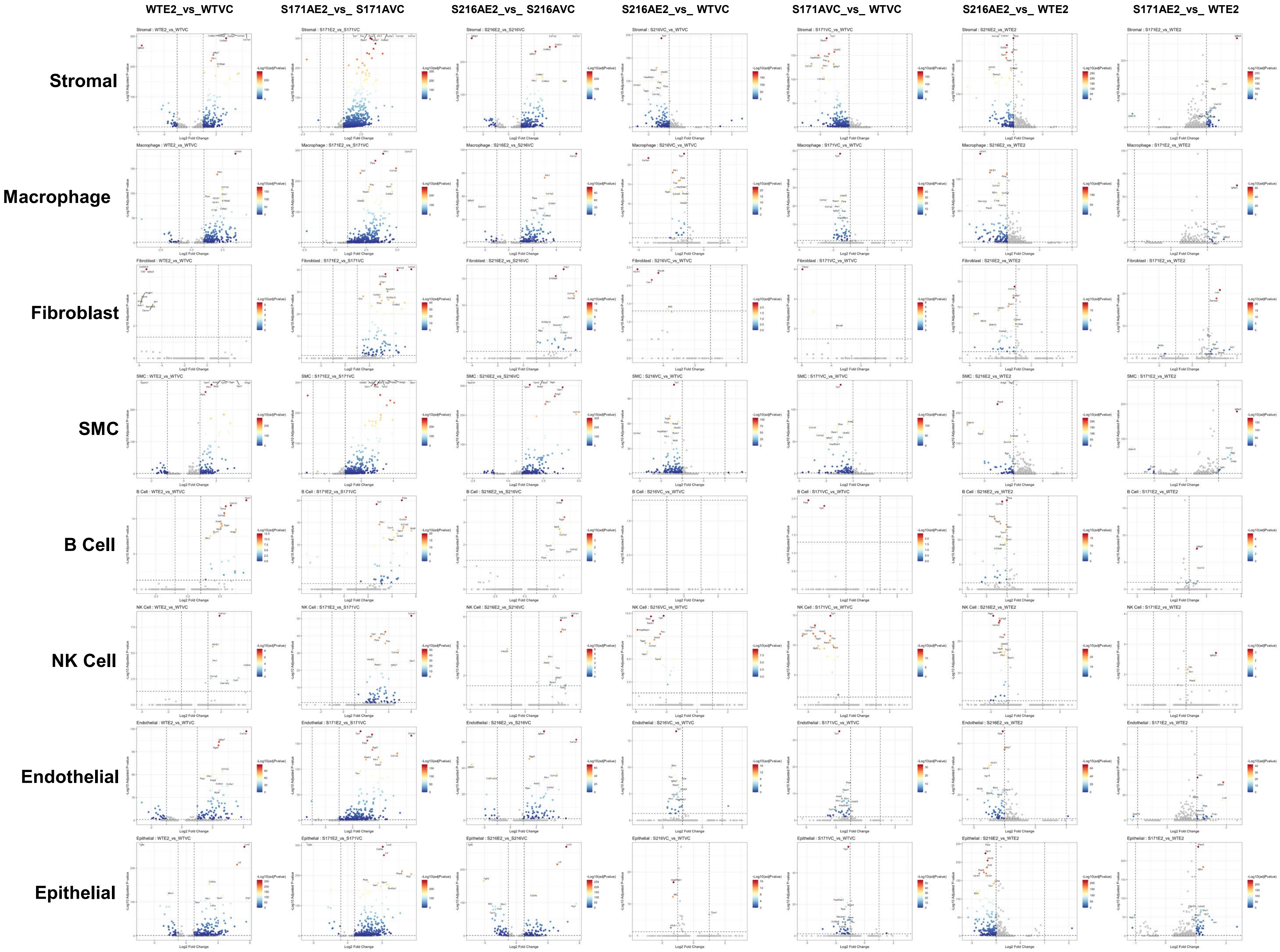
WTE2_vs_WTVC
S171AE2_vs_ S171AVC
S216AE2_vs_ S216AVC
S216AE2_vs_ WTVC
S171AVC_vs_ WTVC
S216AE2_vs_ WTE2
S171AE2_vs_ WTE2
Stromal
Macrophage
Fibroblast
SMC
B Cell
NK Cell
Endothelial
Epithelial

Supplementary Figure 6. Cell Type-Specific Differential Gene Expression Across Genotypes, related to Figure 5.

Volcano plots illustrating transcriptional changes in uterine stromal cells, macrophages, fibroblasts, smooth muscle cells (SMCs), B cells, NK cells, endothelial cells, and epithelial cells between WT, S171A, and S216A mice. Genes with significant expression changes (adjusted $p < 0.05$, log2 fold change > ±1) are highlighted in colored dots, with the scale indicating the log10 adjusted p-value. Non-significant genes are represented by gray dots.

Stromal WTE2_vs_WTVC Up

Biological Process (Gene Ontology) enrichment

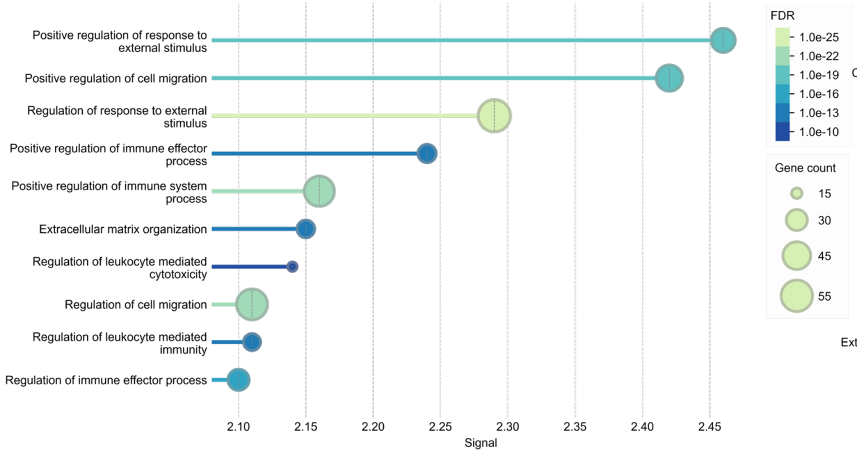

Cellular Component (Gene Ontology) enrichment

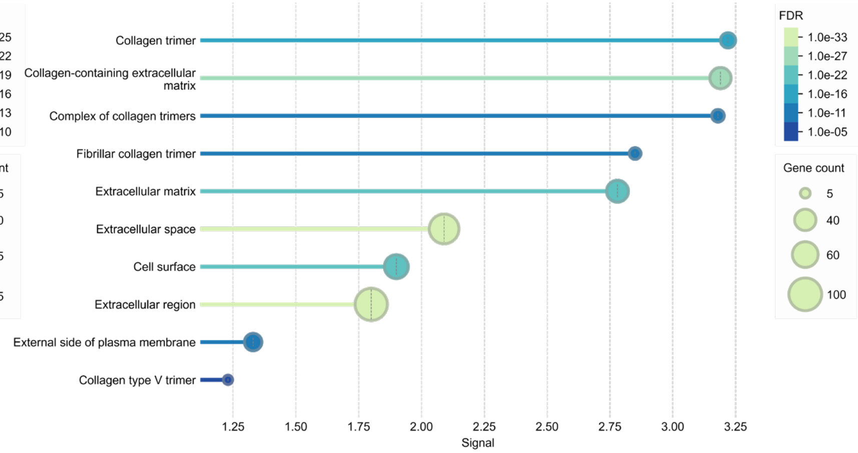

WikiPathways enrichment

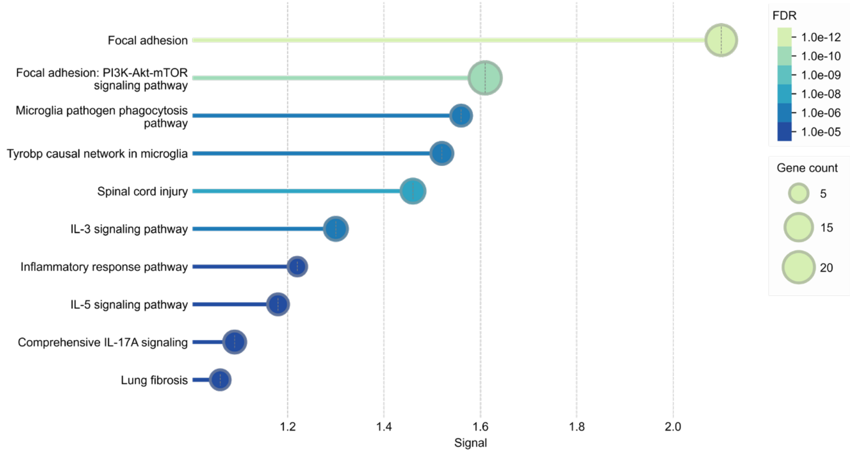

Molecular Function (Gene Ontology) enrichment

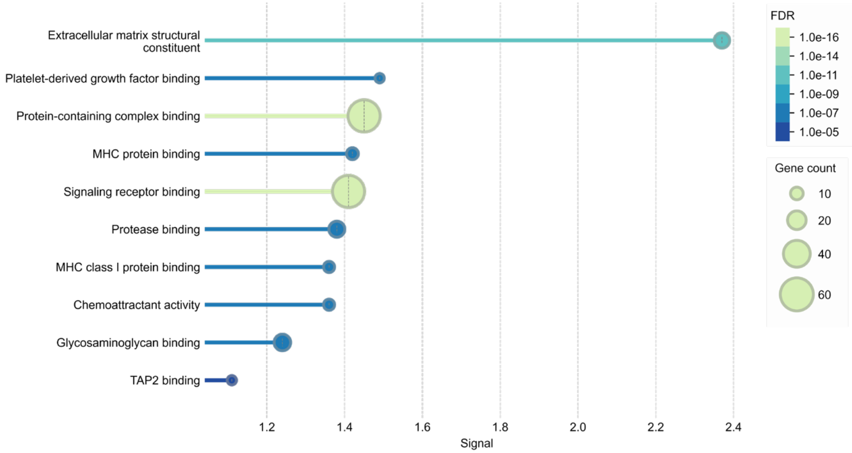

KEGG Pathways enrichment

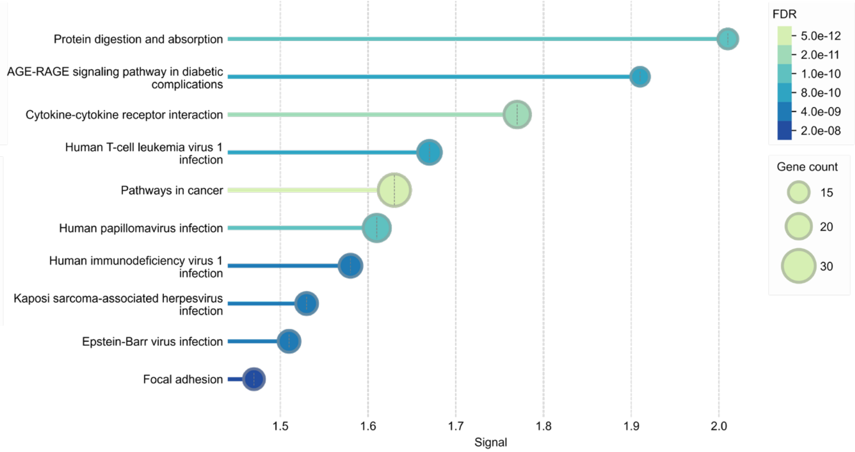

Reactome Pathways enrichment

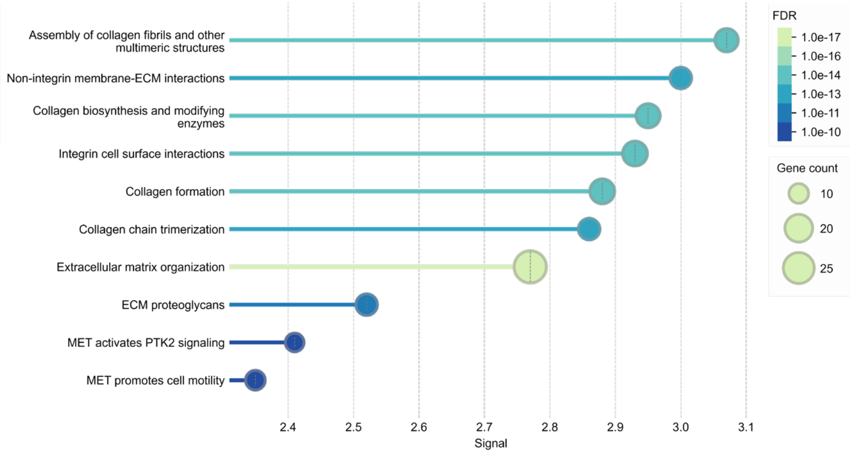

Supplementary Figure 7. Functional Enrichment Analysis of Stromal Cells in WTE2_vs_WTVC, related to Figure 5. Gene ontology (GO) enrichment analysis of biological processes, cellular components, and molecular functions, as well as with KEGG and Reactome pathway enrichment, highlights the functional categories associated with E2-upregulated genes in WT stromal cells. The x-axis represents enrichment signal values, and the size of each dot corresponds to the number of genes in the enriched category. The color scale indicates the false discovery rate (FDR).

Stromal S216E2_vs_WTE2 Down

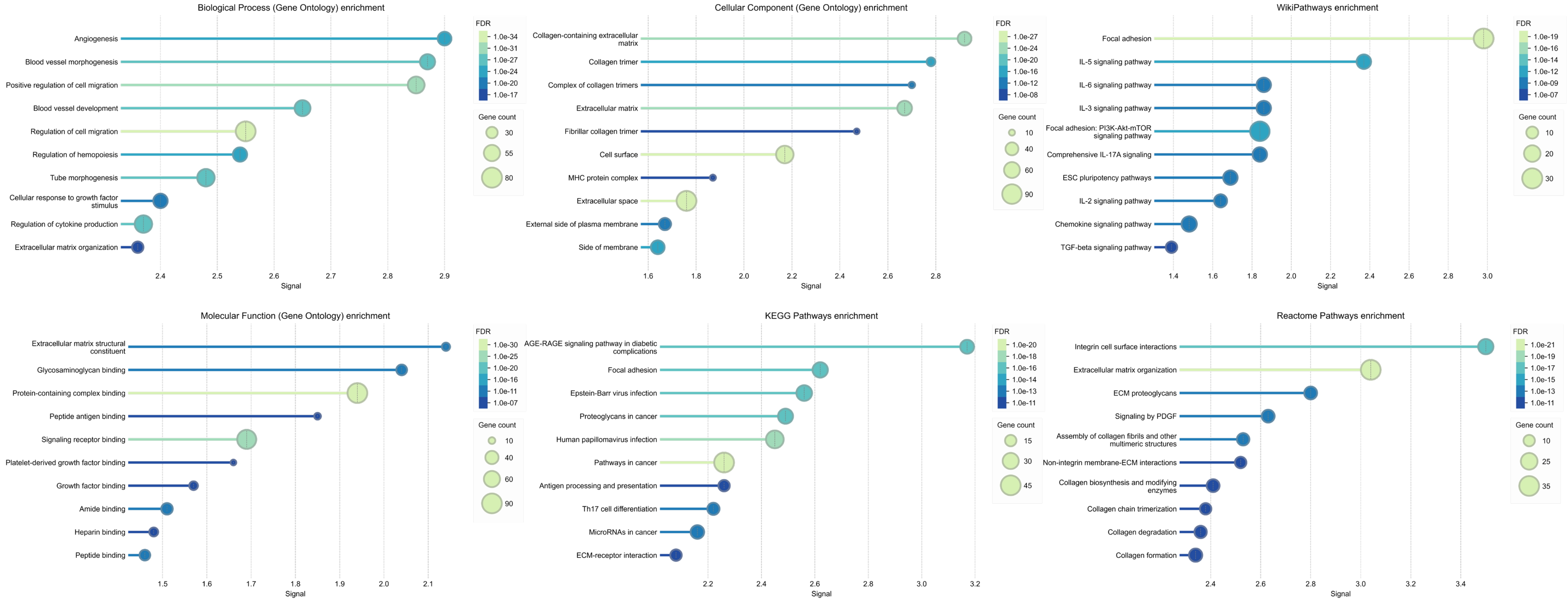

Supplementary Figure 8. Functional Enrichment Analysis of Stromal Cells in S216E2_vs_WTE2, related to Figure 5. Gene ontology (GO) enrichment analysis of biological processes, cellular components, and molecular functions, as well as with KEGG and Reactome pathway enrichment, highlights the functional categories associated with E2-regulated downregulation genes in S216A stromal cells. The x-axis represents enrichment signal values, and the size of each dot corresponds to the number of genes in the enriched category. The color scale indicates the false discovery rate (FDR).

SMC WTE2_vs_WTVC Up

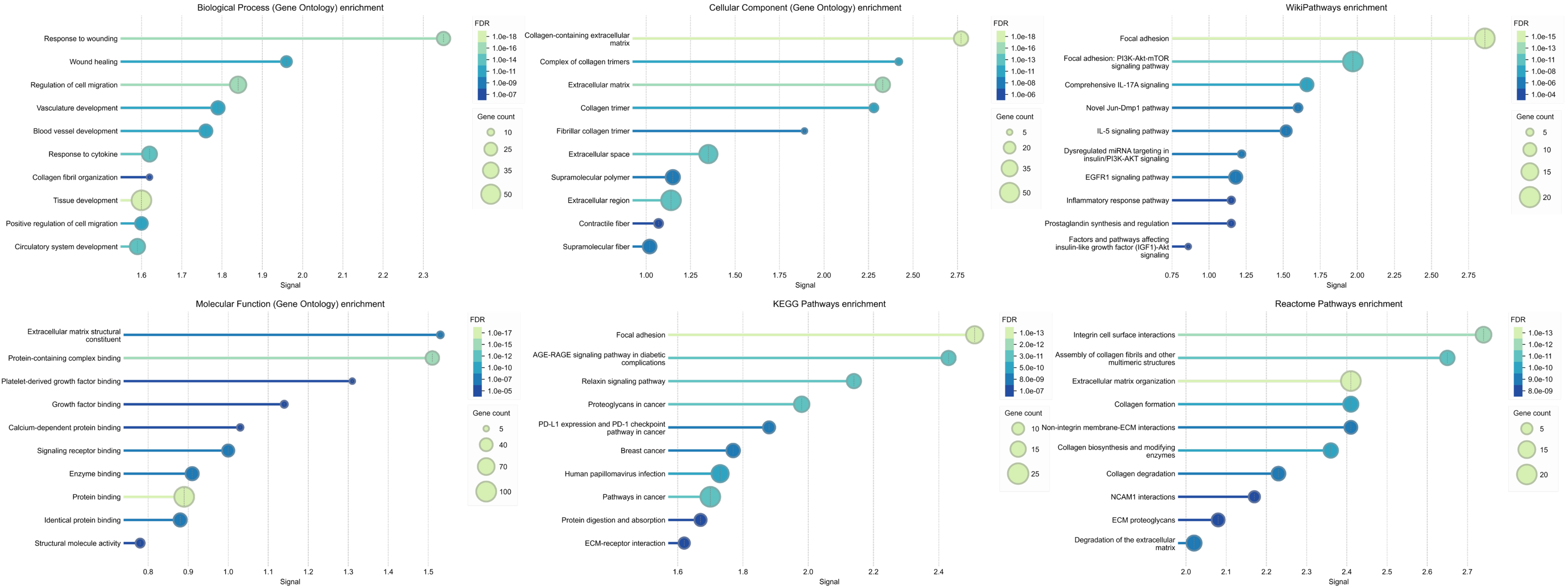

Supplementary Figure 9. Functional Enrichment Analysis of Smooth Muscle Cell (SMC) in WTE2_vs_WTVC, related to Figure 5. Gene ontology (GO) enrichment analysis of biological processes, cellular components, and molecular functions, as well as with KEGG and Reactome pathway enrichment, highlights the functional categories associated with E2-upregulated genes in WT SMC. The x-axis represents enrichment signal values, and the size of each dot corresponds to the number of genes in the enriched category. The color scale indicates the false discovery rate (FDR).

SMC S216E2_vs_WTE2 Down

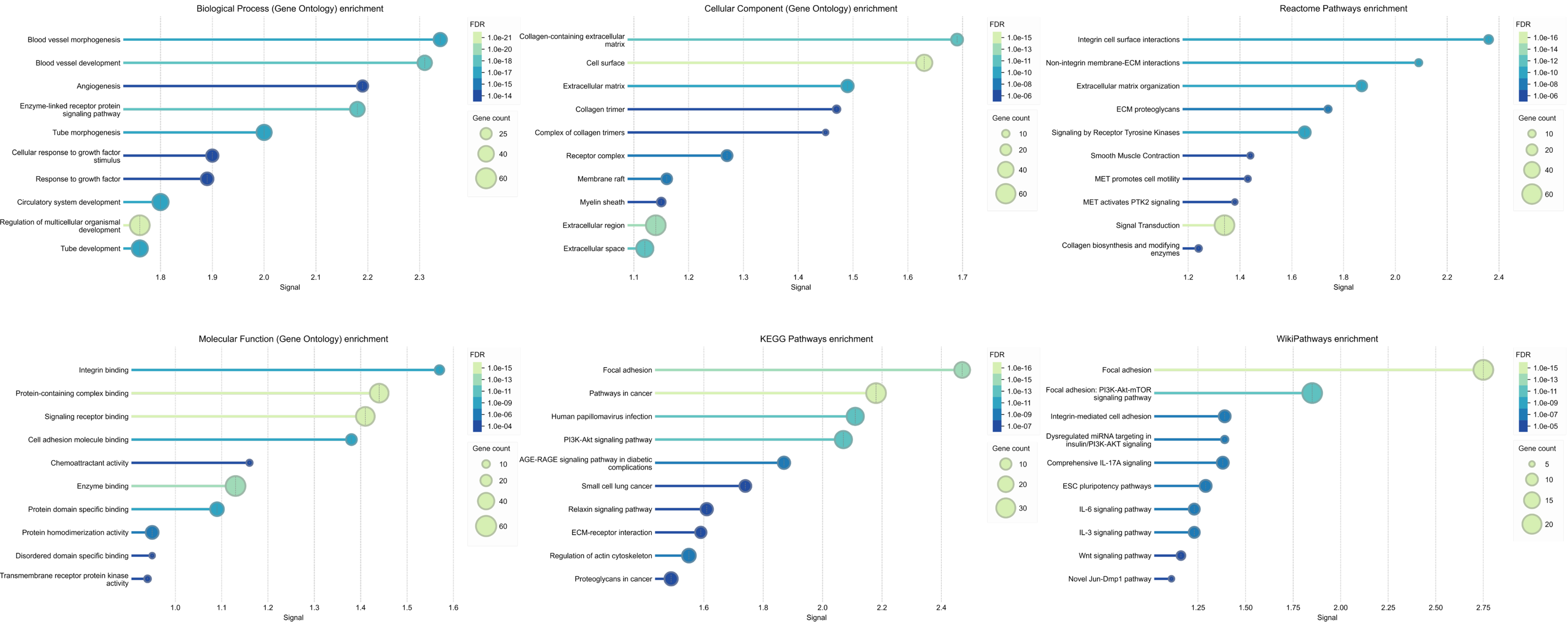

Supplementary Figure 10. Functional Enrichment Analysis of Smooth Muscle Cell (SMC) in S216E2_vs_WTE2, related to Figure 5. Gene ontology (GO) enrichment analysis of biological processes, cellular components, and molecular functions, as well as with KEGG and Reactome pathway enrichment, highlights the functional categories associated with E2-regulated downregulation genes in S216A SMC. The x-axis represents enrichment signal values, and the size of each dot corresponds to the number of genes in the enriched category. The color scale indicates the false discovery rate (FDR).

Epithelial WTE2_vs_WTVC Up

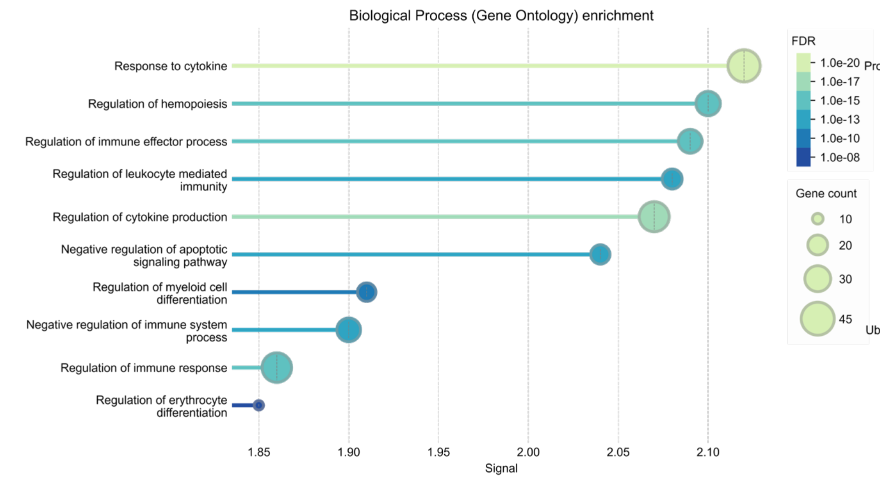

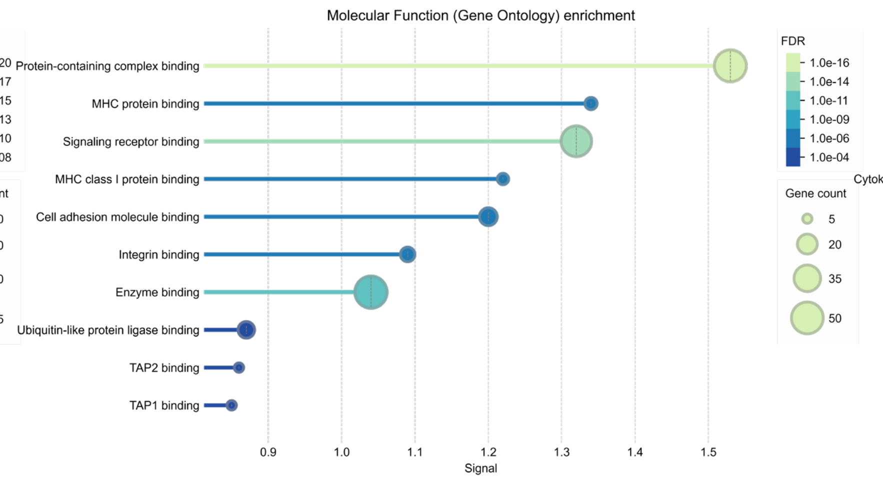

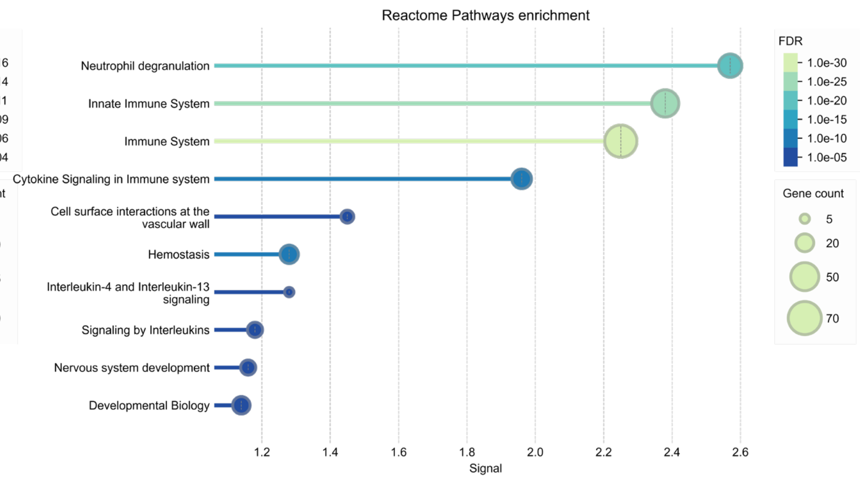

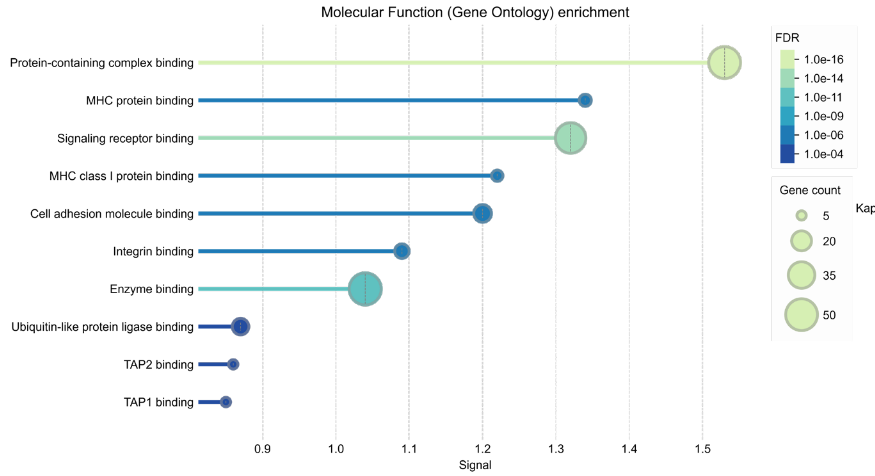

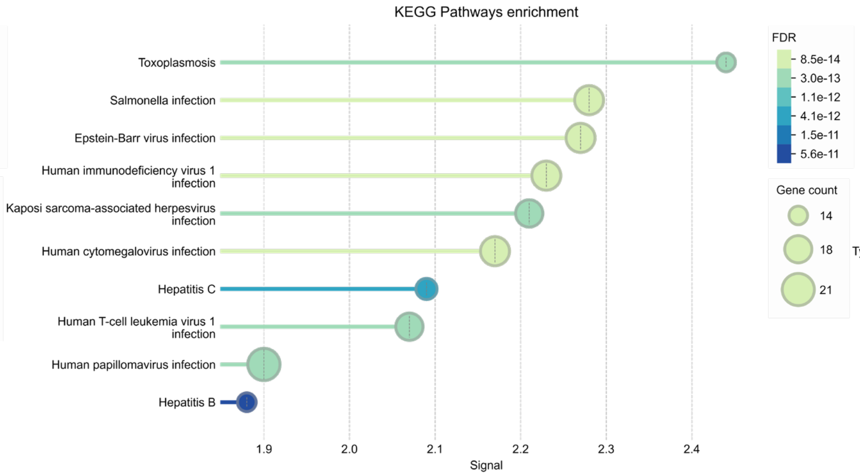

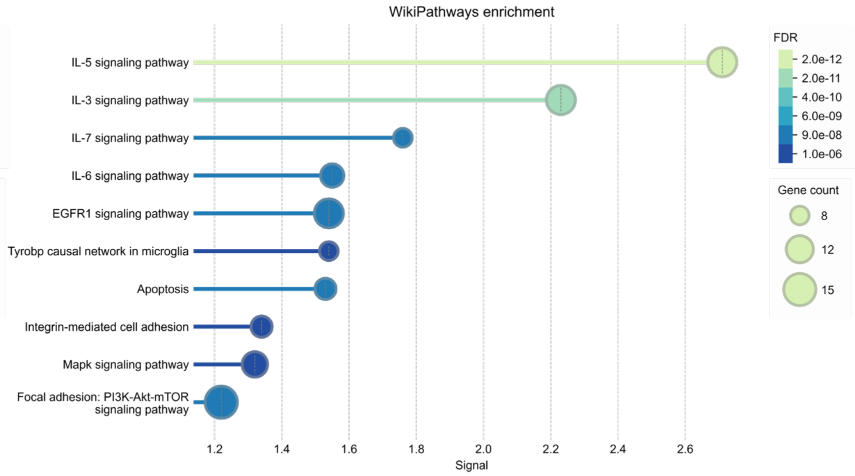

Supplementary Figure 11. Functional Enrichment Analysis of Epithelial Cells in WTE2_vs_WTVC, related to Figure 5. Gene ontology (GO) enrichment analysis of biological processes, cellular components, and molecular functions, as well as with KEGG and Reactome pathway enrichment, highlights the functional categories associated with E2-upregulated genes in WT epithelial cells. The x-axis represents enrichment signal values, and the size of each dot corresponds to the number of genes in the enriched category. The color scale indicates the false discovery rate (FDR).

Epithelial S216E2_vs_WTE2 Down

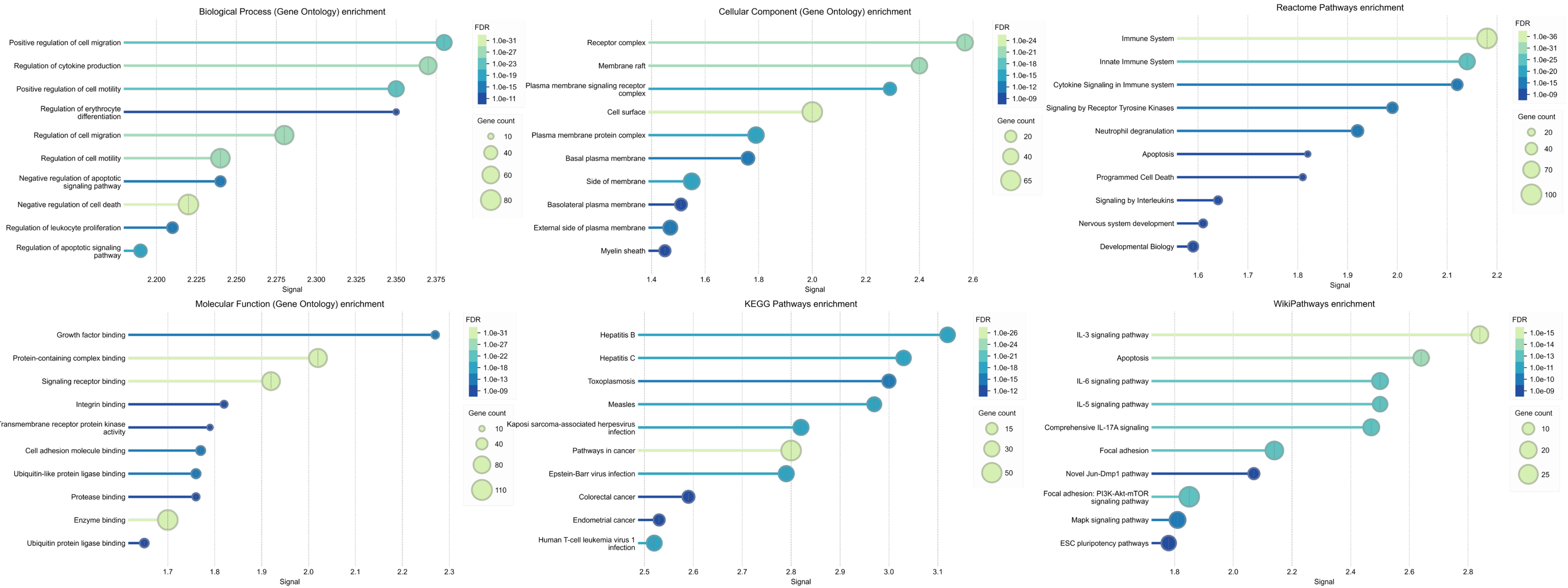

Supplementary Figure 12. Functional Enrichment Analysis of Epithelial Cells in S216E2_vs_WTE2, related to Figure 5. Gene ontology (GO) enrichment analysis of biological processes, cellular components, and molecular functions, as well as with KEGG and Reactome pathway enrichment, highlights the functional categories associated with E2-regulated downregulation genes in S216A epithelial cells. The x-axis represents enrichment signal values, and the size of each dot corresponds to the number of genes in the enriched category. The color scale indicates the false discovery rate (FDR).

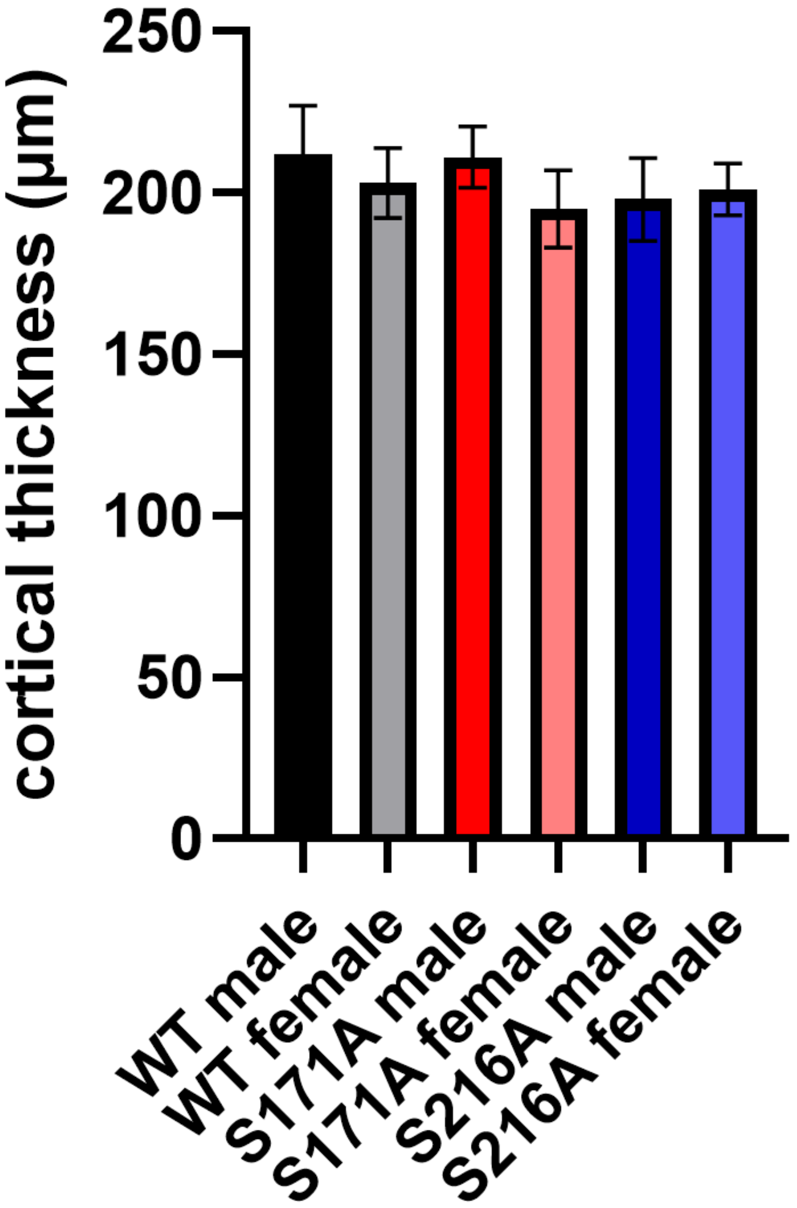

cortical thickness (µm)
250
200
150
100
50
0
WT male
WT female
S171A male
S171A female
S216A male
S216A female

Supplementary Figure 13. Cortical bone thickness in 3-month-old mouse femurs, related to Figure 7. Quantification of femoral cortical bone thickness in 3-month-old WT, S171A, and S216A male and female mice. Data are expressed as mean ± standard deviation (SD); n = 5-7 per group.

References:

1. Sehnal, D., Bittrich, S., Deshpande, M., Svobodová, R., Berka, K., Bazgier, V., Velankar, S., Burley, S.K., Koča, J., and Rose, A.S. (2021). Mol* Viewer: modern web app for 3D visualization and analysis of large biomolecular structures. Nucleic Acids Res *49*, W431-w437. 10.1093/nar/gkab314.
